# A multiplexed, paired-pooled droplet digital PCR assay for detection of SARS-CoV-2 in saliva

**DOI:** 10.1038/s41598-023-29858-5

**Published:** 2023-02-22

**Authors:** Kaitlyn Wagner, Phil Fox, Elizabeth Gordon, Westen Hahn, Kenzie Olsen, Alex Markham, Dylan Buglewicz, Platon Selemenakis, Avery Lessard, Daniella Goldstein, Alissa Threatt, Luke Davis, Jake Miller-Dawson, Halie Stockett, Kristin Rugh, Houston Turner, Michelle Remias, Maggie Williams, Jorge Chavez, Gabriel Galindo, Charlotte Cialek, Amanda Koch, Alex Fout, Bailey Fosdick, Bettina Broeckling, Mark D. Zabel

**Affiliations:** 1 Prion Research Center, Department of Microbiology, Immunology and Pathology, College of Veterinary Medicine and Biomedical Sciences, Fort Collins, USA; 2 Department of Statistics, Colorado State University, Fort Collins, CO 80523 USA; 3 Colorado State University, Fort Collins, CO 80523 USA; 4 Colorado School of Public Health, University of Colorado Anschutz Medical Campus, Aurora, USA

**Keywords:** Diseases, Infectious diseases, Health care, Diagnosis, Microbiology

## Abstract

In response to the SARS-CoV-2 pandemic, we developed a multiplexed, paired-pool droplet digital PCR (MP4) screening assay. Key features of our assay are the use of minimally processed saliva, 8-sample paired pools, and reverse-transcription droplet digital PCR (RT-ddPCR) targeting the SARS-CoV-2 nucleocapsid gene. The limit of detection was determined to be 2 and 12 copies per µl for individual and pooled samples, respectively. Using the MP4 assay, we routinely processed over 1,000 samples a day with a 24-h turnaround time and over the course of 17 months, screened over 250,000 saliva samples. Modeling studies showed that the efficiency of 8-sample pools was reduced with increased viral prevalence and that this could be mitigated by using 4-sample pools. We also present a strategy for, and modeling data supporting, the creation of a third paired pool as an additional strategy to employ under high viral prevalence.

## Introduction

The worldwide Covid-19 pandemic presented enormous challenges to multi-congregate settings such as schools and universities, including how to maintain robust educational programs while ensuring the safety of their communities^1^. Universities weighed a variety of concerns when formulating plans for the Fall semester (typically August-December) of 2020, a time when most people were unvaccinated^2–4^. Reopening plans varied from fully remote to fully in-person as well as approaches in between^5^. To facilitate efforts to return to in-person learning, routine screening was proposed to identify infected, asymptomatic individuals and, in combination with other interventions, mitigate the spread of SARS-CoV-2^6^. Many universities developed or implemented screening assays, using a variety of sample types (saliva vs nasal swabs), sample processing (minimal vs RNA extraction), efficiency strategies (pooled vs unpooled), and platforms (qPCR, RT-LAMP, antigen tests), although the majority relied on qPCR^7–22^. A recent analysis of 1,400 institutions of higher education (IHE) showed that the success of these screening efforts extended to their housed counties, as those with IHEs that conducted widespread testing had fewer hospitalizations and deaths^23^.

We developed a saliva-based screening assay for Colorado State University, implemented in October of 2020, as a key component of the university’s effort to safely reopen the campus to in-person learning. Our goal was to implement an accurate, high-throughput (~ 1,000 samples a day), saliva-based screening assay with next-day turnaround time and sufficient sensitivity to detect people who may be infected and direct them to an FDA-approved test. Here we report on the SARS-CoV-2 nucleic acid amplification assay we developed as part of the university’s response to the pandemic, which we have named the MP4 assay for multiplex, paired-pooled ddPCR.

## Results

### Description of the MP4 assay

**Figure 1:**
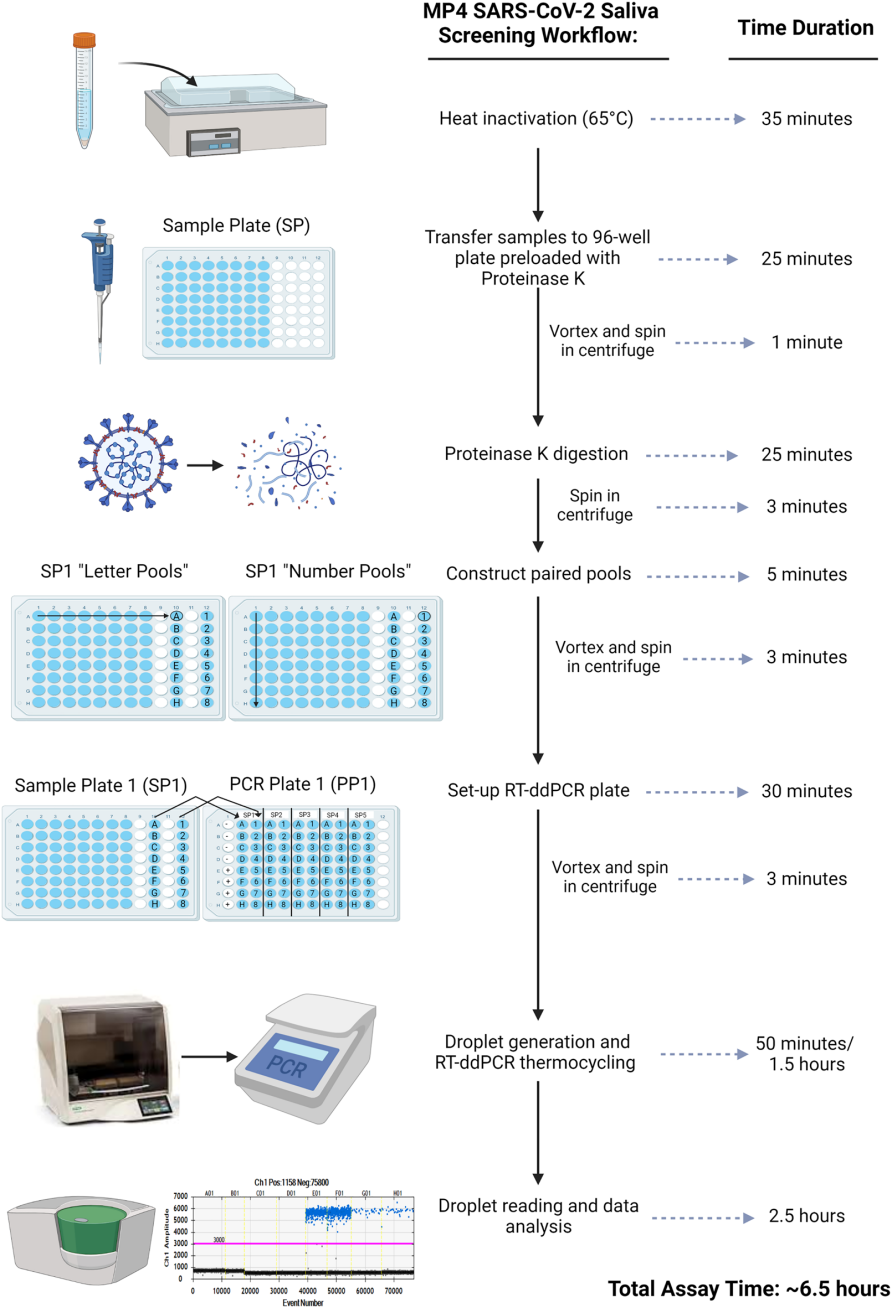
MP4 screening assay overview. Saliva samples from asymptomatic adults were collected, heat inactivated, and individually pipetted into columns 1–8 of a 96-well sample plate (SP) preloaded with proteinase K. Following a brief incubation and PK inactivation step, two paired pools (letter and number) were created as outlined in Materials and Methods. Pooled samples were transferred to a PCR plate (PP) containing RT-ddPCR master mix. Pooled samples for up to 5 SPs (2 columns per SP) plus one column of control samples were run per PP. After droplet generation in an Automated Droplet Generator, droplets were transferred to a thermocycler for cDNA synthesis and amplification, and then droplet fluorescence was read in a Droplet Reader (FAM/HEX channels). Thresholds for delineating positive and negative droplets were set using control samples. Positive pools (≥ 3 N1 droplets) were flagged for deconvolution and confirmatory testing of individual samples. Figure was created with BioRender.com.

An overview of the MP4 screening assay is presented in Fig. 1. Saliva, collected in barcoded tubes across campus (Supplementary Fig. s1), was brought to the lab by couriers and immediately placed in a 65 °C water bath for 35 min to inactivate the SARS-CoV-2 virus. Sample barcodes were then scanned into an electronic sample spreadsheet. In a biosafety cabinet, saliva samples were manually pipetted into the first 8 columns of a 96 well plate preloaded with Proteinase K (PK) for a maximum of 64 samples per plate. Following a brief incubation step, PK was heat inactivated and 2 paired pools were constructed.

As discussed in more detail below, we implemented an 8-sample paired pooling strategy (also known as 2D pooling^24^), to reduce reagent consumption, assay cost, and time. First, a “letter” pool was constructed by horizontally pooling samples in rows A-H. Second, a “number” pool was constructed by vertically pooling samples in columns 1–8. This pooling strategy reduced the number of PCR reactions fourfold, from 64 to 16 while running each sample in duplicate, since each was present in two distinct pools.

For SARS-CoV-2 detection, we used the RT-ddPCR workflow optimized and validated by BioRad in combination with primers developed by the CDC. We multiplexed the N1 primers and probes, which target the SARS-CoV-2 nucleocapsid gene; and RP primers and probes, which target the human RNase P gene and act as an internal control that confirms a human sample. We also confirmed successful mulitiplexing of N1 and N2 primers and probes that also target the nucleocapsid gene. Linearity of the MP4 assay was evaluated using serially diluted viral RNA, which demonstrated a broad linear range of 0.29– 7,300 copies per μl in the RT-ddPCR reaction for N1 (R^2^: 0.9990) (Supplementary Fig. s2), comparing favorably to that reported by BioRad (0.5- 6,000 copies per μl).

RT-ddPCR reactions were constructed in 96 well plates, such that paired pools from up to 5 sample plates representing 320 individual samples could be run per PCR plate (Fig. 1). We also included one column (8 samples) of controls on every plate, run in duplicate: SARS-CoV-2 pooled negative saliva, water, and high (375 copies per µl) and low (12.5 copies per µl) concentrations of a SARS-CoV-2 heat-inactivated virus. Droplets were generated in an Automatic Droplet Generator, RT and end-point PCR cycling were performed in a C1000 thermocycler, and droplet fluorescence was read in a Droplet Reader. The time from sample receipt to the initiation of droplet reading took ~ 5–6 h (Fig. 1; see Supplementary Fig. s3 for an example workflow and staffing).

The droplet reader reads each droplet for fluorescence in two channels (FAM & HEX), and after applying thresholds, 4 populations of droplets can be distinguished: negative droplets, droplets positive for N1 (FAM) only, droplets positive for RP (HEX) only, and droplets positive for both targets. (Supplementary Fig. s4). Pools with ≥ 3 N1 droplets were scored as positive. Following deconvolution of the pools (described below), individual samples were identified for subsequent testing (reruns). With low positivity rates and approximately 1,000 saliva samples collected per day, reruns were typically completed, and results reported, by noon of the day following sample submission.

### Deconvolution of positive pools

**Figure 2:**
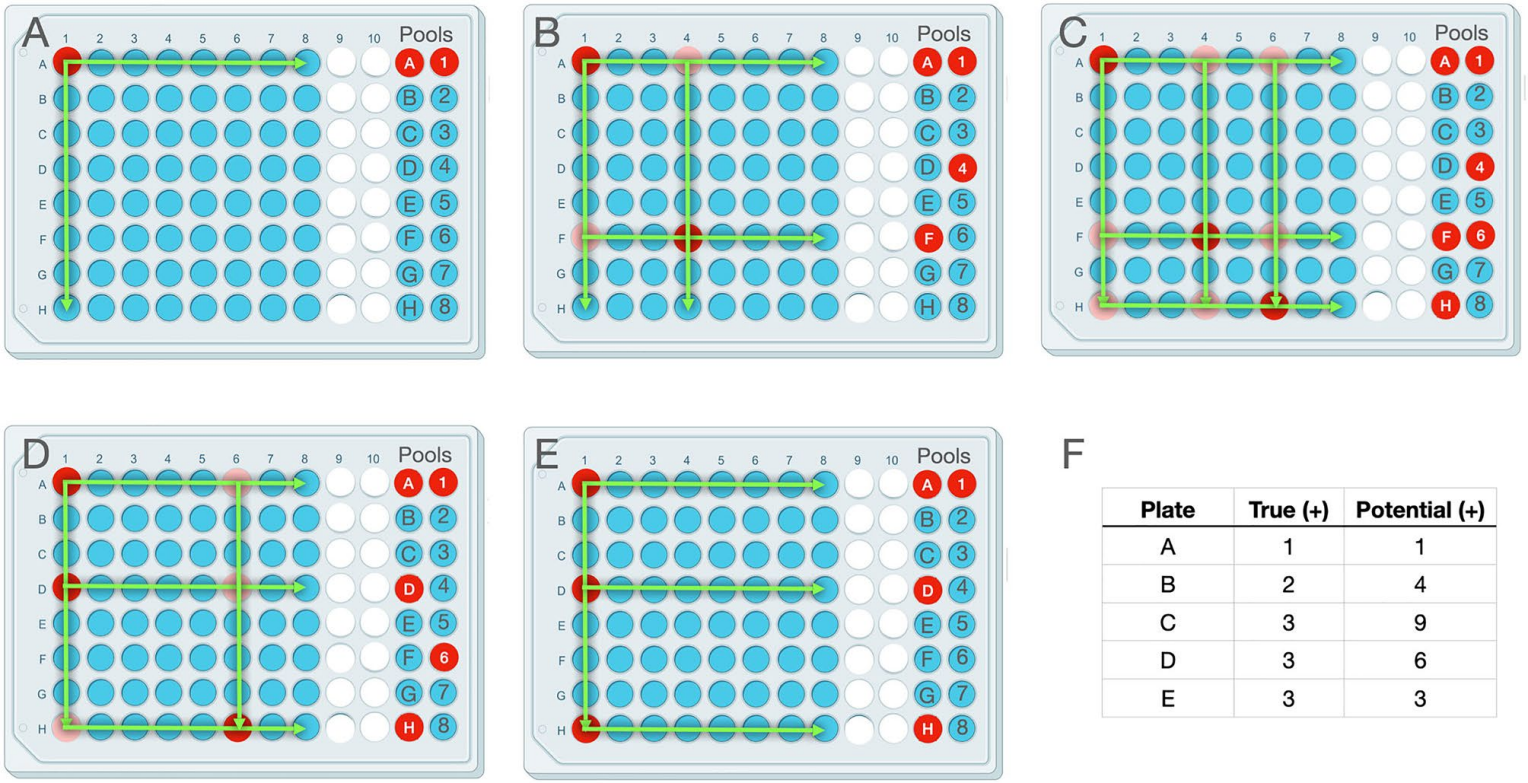
Deconvolution of positive individual samples by decoding paired pools. Sample deconvolution scenarios for plates containing one **(A)**, two **(B)**, or three **(C-E)** positive samples per 64-sample plate. Columns 1–8 represent individual samples, with positive samples shown in red. Pooled samples are shown on the right, with positive pools indicated in red. (**A**) When the positivity rate is low (1/64; < 2%), single positives can be identified unambiguously. (**B-E**) More than one positive sample per plate (> 3%) requires deconvoluting true positives (red) from potential positives (red and pink). (**F**) As the number of true positives (equal to the (greater of) the number of positive pools from either letter or number pools) increase on a sample plate, the number of potential positives (equal to the product of the number of positive letter pools times the number of positive number pools) increases. In rarer cases, a pool may contain several positives, which are surprisingly easy to identify **(D, E)**.

As outlined above and detailed in the Materials and Methods, we created paired pools, each containing 8 individual saliva samples, with each saliva sample present in 2 unique pools. Positive pools containing ≥ 3 N1 droplets were considered positive, and after a deconvolution step, individual samples were identified for additional testing. Figure 2 presents scenarios for deconvolution of 1, 2, or 3 positive samples per plate assuming perfect test sensitivity. If only one positive sample is present in the plate (< 2% positivity), the intersection of the positive pools unambiguously identifies the positive sample (Fig. 2A). For plates with more than one positive sample (> 3%, Fig. 2B-E), the intersections identify “potential positive” samples that must be run individually to discriminate the true positives. The number of potential positive samples per plate not only depends on the number of true positive samples, but also on their spatial arrangement. As shown in Fig. 2C-E, which presents 3 different scenarios for plates with 3 positive samples per plate, the number of potential positive samples requiring reruns ranged from 3–9, depending on how those samples are arranged on the plate and therefore represented in the pools (Fig. 2F).

**Figure 3:**
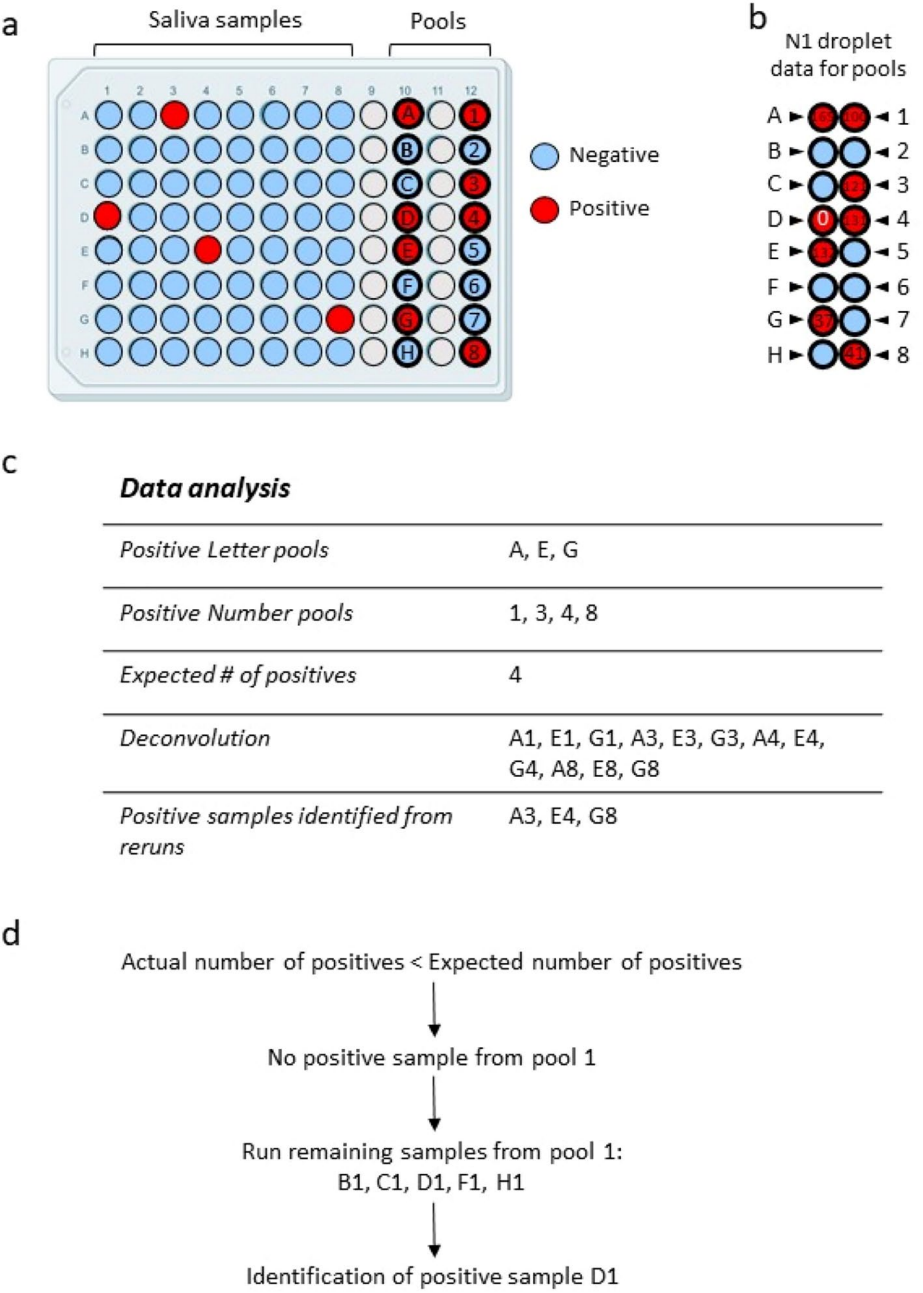
Example dataset and workflow for identification of a positive sample with only one positive pool. (**a)** Schematic of the sample plate with positive (red) and negative (blue) samples and pools (bold outline). (**b)** N1 droplet counts for the pools. Pool D, which should have been positive, is indicated in red. (**c)** Data analysis including the positive pools, expected number of positive samples, deconvolution, and rerun data. (**d)** Outline of workflow when the actual number of positives (3) was less than expected, resulting in the identification of positive sample D1.

In the scenario in which only one individual letter or number pool is positive (*ex*: pool B), with no positive matching pool (*ex*: pools 1–8 are all negative), the recourse is to re-run all 8 samples from the positive pool (*ex*: B1, B2, B3, B4, B5, B6, B7, B8). Figure 3 presents a slightly more complicated scenario, taken from actual data, in which a positive pool is missed in a plate with more than one positive sample. In this example, the sample plate had 4 positive samples (red), with the expected positive pools indicated by bold-outlined red circles in Fig. 3a. Pool D, which should have been positive, was called negative due to the absence of measured N1 droplets (Fig. 3b) and thereby omitted from the subsequent deconvolution and rerun steps (Fig. 3c). In the MP4 assay, we expect the number of true positives to be the (greater) number of positive pools from either letter or number pools. Because there were 3 positive letter pools, and 4 positive number pools, we expect to find 4 positive individual samples from this plate. The fact that we detected an unequal number of positive letter and number pools is not unusual because two positives can be present in the same letter or number pool (*ex*: A1 and A3 are both in pool A, but in different number pools). When the samples identified from the deconvolution step were tested, only 3 positive samples were identified. Because there were no positives identified from pool 1, the remaining samples from pool 1 were rerun and sample D1 was identified (Fig. 3d).

### Limit of detection

**Figure 4:**
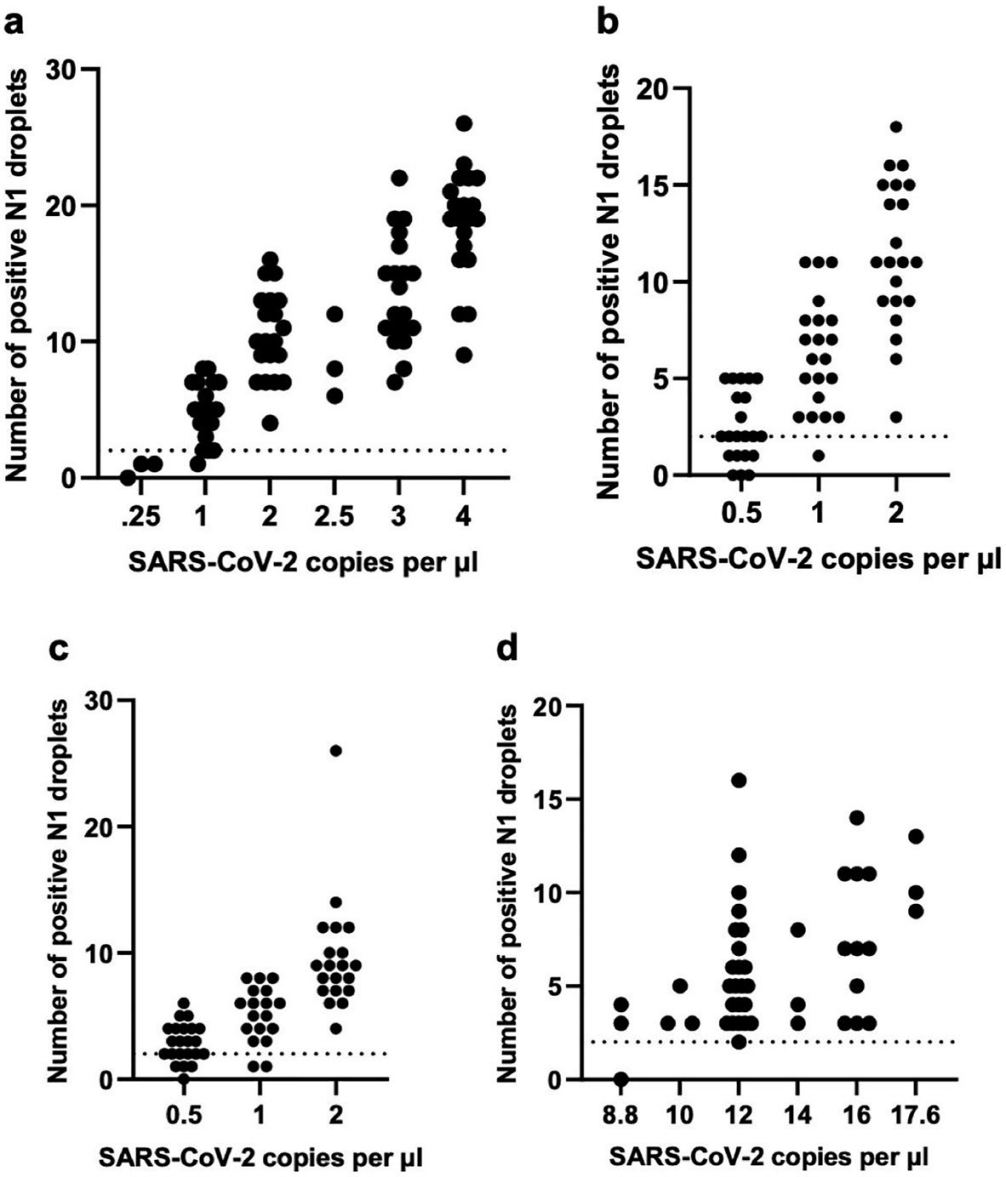
Determination of the LOD for MP4 assay**.** LOD was determined in single-sample **(a-c)** and pooled **(d)** assays using three different SARS-CoV-2 virus sources. LOD for QE-extracted virus in water **(a)** and purified viral RNA diluted in water **(b)** and were determined to be 2 and 1 SARS-CoV-2 copies per µl, respectively. The LOD in single-sample **(c)** and pooled **(d)** assays in which negative saliva was spiked with a positive saliva sample of known virus concentration was determined to be 2 and 12 SARS-CoV-2 copies per µl, respectively. Experiments in (c,d) were performed using PK-digested saliva according to the MP4 assay. Data points above the dotted line (set at 2 copies per µl) meet our threshold of ≥ 3 positive N1 droplets. LOD is represented as SARS-CoV-2 copies per µl in the starting sample and was defined as the lowest concentration of virus in which ≥ 95% of replicates (n ≥ 20) were scored as positive.

We determined the limit of detection of the MP4 assay using three different SARS-CoV-2 virus sources (Fig. 4). For each viral source, LOD was determined by serial dilution and defined as the lowest concentration of virus in the starting sample in which ≥ 95% of replicates (n ≥ 20) were scored as positive (≥ 3 N1 droplets). Initially, we relied on a heat-inactivated virus obtained from BEI Resources, which in our hands was extremely sensitive to the dilution and extraction steps. For instance, when we spiked the virus in either negative saliva or viral transport media (VTM^25^) followed by PK digestion and RT-ddPCR, we were only able to quantify 5 or 12.8%, respectively, of the amount of virus predicted based on concentrations provided by the manufacturer. Initial LOD studies using the BEI virus diluted in negative saliva and extracted with PK gave an LOD of 11 SARS-CoV-2 copies per µl (Supplementary Fig. s5). Extraction of this heat-inactivated virus using QE solution gave much better results, leading to quantification of 67% of the expected amount of virus. Using this QE workflow, we determined the LOD to be 2 SARS-CoV-2 copies per µl (Fig. 4a). We tried to circumvent the problems we had with the heat-inactivated virus from BEI by preparing our own reference materials, using RT-ddPCR to quantify purified viral RNA and a positive saliva sample, but we note that this strategy was not ideal as the quantification did not take into account the efficiency of the extraction, RT, or ddPCR steps, all of which could significantly alter the final quantification^26^. With these considerations in mind, we determined the LOD for SARS-CoV-2 viral RNA serially diluted in water to be 1 SARS-CoV-2 copy per µl (Fig. 4b). The LOD for a positive saliva sample diluted in pooled SARS-CoV-2 negative saliva and processed using PK as described for the MP4 assay was 2 SARS-CoV-2 copies per µl (Fig. 4c). Based on these 3 different SARS-CoV-2 reference materials we report a LOD for the MP4 assay of 2 SARS-CoV-2 copies per µl in the starting sample. We also experimentally determined the LOD for pooled saliva samples (also processed according to the MP4 assay) using the quantified positive saliva sample and pools of 8 in which one sample was positive. This LOD was determined to be 12 SARS-CoV-2 copies per µL (Fig. 4d), slightly lower than predicted (16 SARS-CoV-2 copies per µL) based on the LOD for individual samples (2 SARS-CoV-2 copies per µL).

### Comparison to FDA-approved test

Individuals whose samples tested positive by our MP4 assay were directed to take an FDA-authorized anterior nasal swab (ANS) test for confirmation. While most of these ANS samples also tested positive, we do not know exact correlation for all 250,000 samples we screened. To determine how well the MP4 saliva assay performed for detection of SARS-CoV-2 individuals, saliva and self-collected ANS samples were contemporaneously obtained from individuals on campus reporting Covid-19 symptoms and/or close contact with Covid-positive individual(s) and tested in parallel. Saliva samples were tested according to our MP4 assay; ANS samples were tested by a commercial diagnostic laboratory (Biodesix, Boulder, CO) using an FDA-approved ddPCR SARS-CoV-2 test. The MP4 screening assay correctly identified all 26 saliva samples with matching positive ANS samples (26/26 = 100% positive percent agreement (95% CI 86.8–100%)). Saliva from 32 of 34 ANS negative individuals tested negative by the MP4 assay (32/34 = 94.1% negative percent agreement (95% CI 80.3–99.3%)). The MP4 test thus identified 2 additional saliva samples as positive from individuals with a negative ANS. These two samples had 244 and 271 N1 droplets in our assay, a level that is ~ 2-orders of magnitude higher than our LOD, and thus unequivocal positives. Symptoms were reported by one of these individuals, the other was asymptomatic but reported a close contact.

### Example experiment

**Figure 5:**
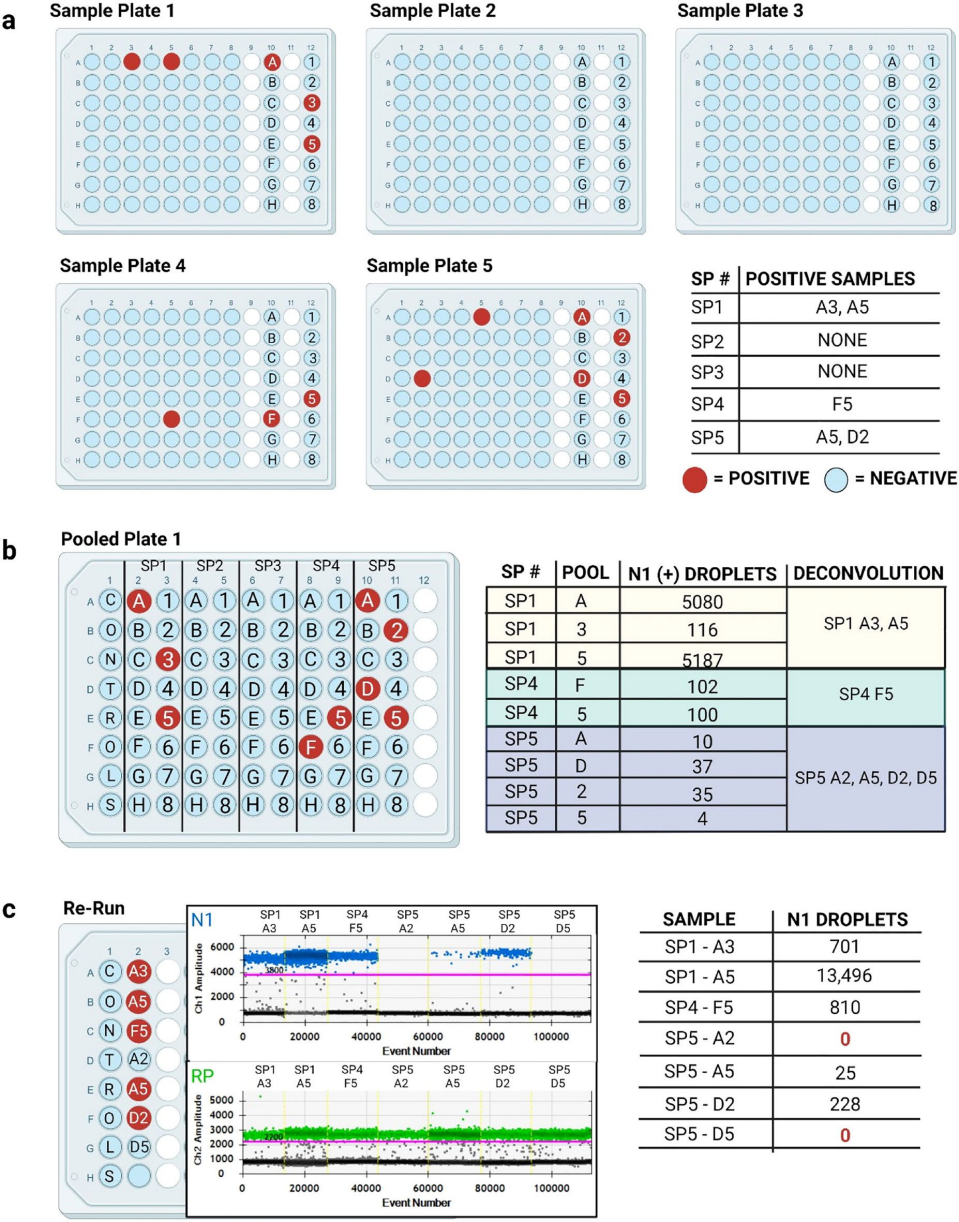
Representative example of data from a typical PCR plate and subsequent confirmatory testing. (**a)** Schematic of 5 sample plates (SPs) containing 0–2 positive samples (red) identified by the MP4 screening assay. Negative samples are shown in light blue. (**b)** Schematic of the PCR plate containing pooled samples from SP1-5. N1 droplet numbers for each positive pool as well as deconvolution results are shown in the table. (**c)** Schematic of the re-run plate with individual samples identified in B. Quantasoft charts showing fluorescent amplitudes of N1 (top, blue) and RP (bottom, green) droplets for each sample are shown. Thresholds distinguishing positive from negative droplets are shown as pink lines. N1 droplet numbers for the individual samples are shown in the table.

To illustrate the utility of the MP4 assay, Fig. 5 presents data from one PCR plate that was run in February of 2022 as a representative example. This PCR plate included 80 pooled samples resulting from 320 individual samples. The number of positive samples we ultimately detected on the sample plates ranged from 0–2 out of 64 for a maximum positivity rate of 3.1% (Fig. 5a). Pools scored as positive had 4- 5,187 N1 droplets and we identified 7 samples for individual testing (reruns) (Fig. 5b). SP1 had 1 positive letter pool and 2 positive number pools. Both samples identified from the positive pools were confirmed positive after deconvolution (Fig. 5c). SP4 had 1 positive letter pool and 1 positive number pool, and we confirmed the sample identified from these pools to be positive after deconvolution. SP5 had 2 positive letter pools and 2 positive number pools, and 4 samples identified for confirmatory testing. Only two of these samples were confirmed to be positive, as expected.

### Comparisons between pooled and individual SARS-CoV-2 positive saliva samples

**Figure 6:**
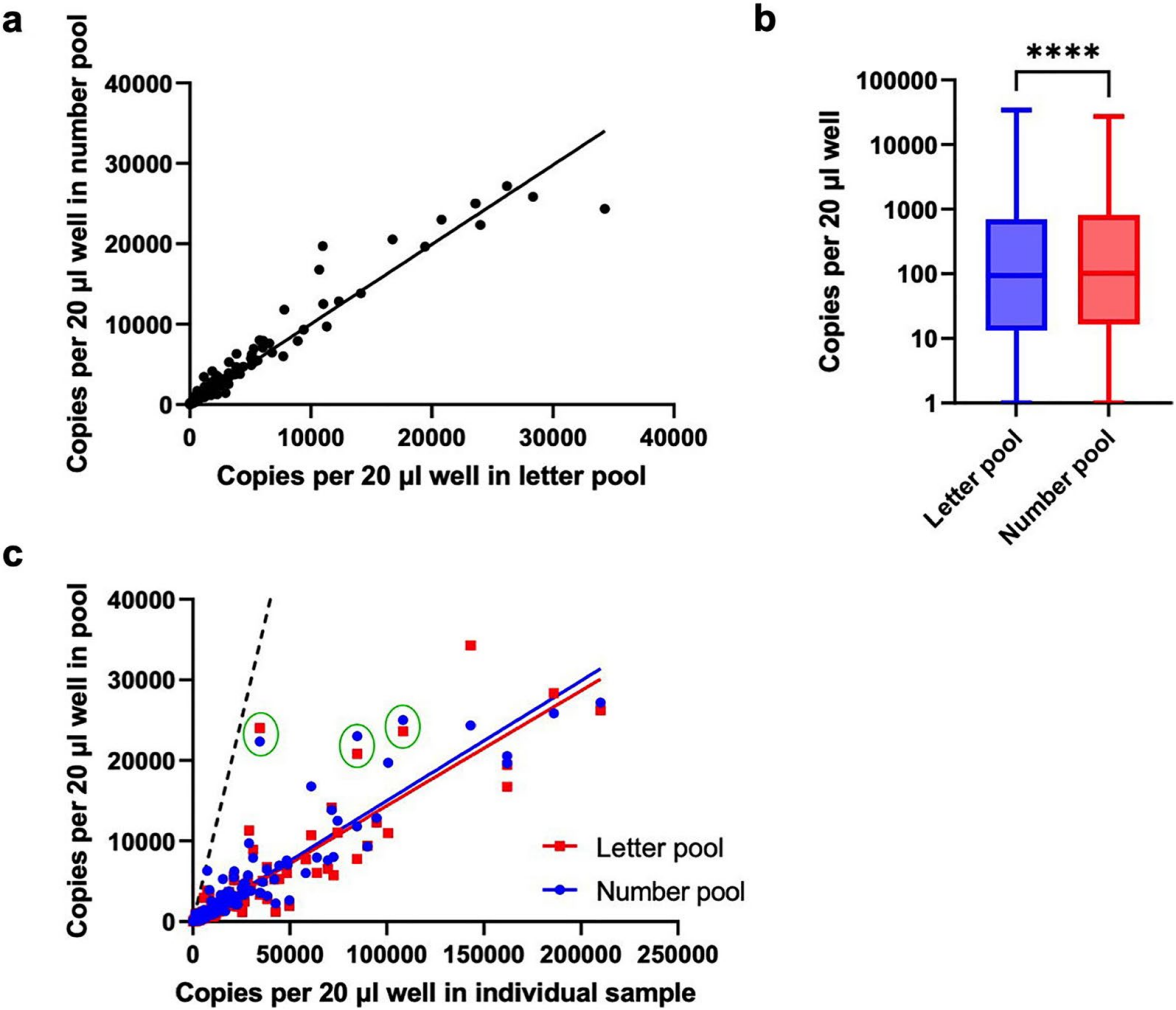
Pooled and individual detection of SARS-CoV-2 in saliva collected during the Fall 2021 semester (August 23- December 17). Pools with more than one positive sample and samples with no negative droplets were removed before analysis. (**a)** Concordance between letter and number pools. Pearson’s correlation coefficient, r = 0.974 (p < 0.0001). (**b)** Box and whisker plots of copies per reaction for letter and number pools. Data are plotted on a log_10_ scale. Boxes extend from the 25th to 75th percentiles with median indicated by a line. The letter versus number pools comparison had a median of differences of 4. Asterisks (****) denote a p-value < 0.0001 as determined using Wilcoxon matched-pairs signed rank test. **(c)** Plot of pooled versus individual copies per reaction for letter (red) and number (blue) pools. Pearson’s r = 0.907 (p < 0.0001) and 0.929 (p < 0.0001) for letter and number pools, respectively. Green circles indicate paired pool samples that deviate from the trendline. The dashed line indicates the line of identity (x = y).

In analyzing data from the Fall semester of 2021 (August 23- December 17), in which we identified 385 positive saliva samples originating from pools with only one positive per pool, we observed a high concordance between the letter and number pools (Pearson’s r = 0.974, (p < 0.0001)), (Fig. 6a), indicating a high degree of technical precision in the two separate pooling steps. Despite this concordance, we detected slightly higher viral copy numbers in number versus letter pools (median of differences of 4 copies per µl) (Fig. 6b). The relationship between pools versus the individual samples also indicated a high degree of linearity (Pearson’s r = 0.907 (p < 0.0001) and 0.929 (p < 0.0001) for letter and number pools, respectively) with viral copy numbers in the pools 6.7- 7.0-fold less than the individual sample, close to the expected eightfold difference (Fig. 6c).

We observed a wide range of viral loads in individual positive samples, ranging from 3- 210,000 copies per reaction (mean value of 9,440; 95% CI for mean 6,850- 12,030). This translates to approximately 0.4- 28,747 copies per µl in the saliva sample. The frequency distribution was skewed toward the low end of this range (Supplementary Fig. s6), with a median value of 519 copies per reaction (25 and 75% percentiles of 92 and 5160 copies per reaction, respectively), translating to 71 copies per µl in the saliva sample.

### 4-versus 8-sample pooling

**Figure 7:**
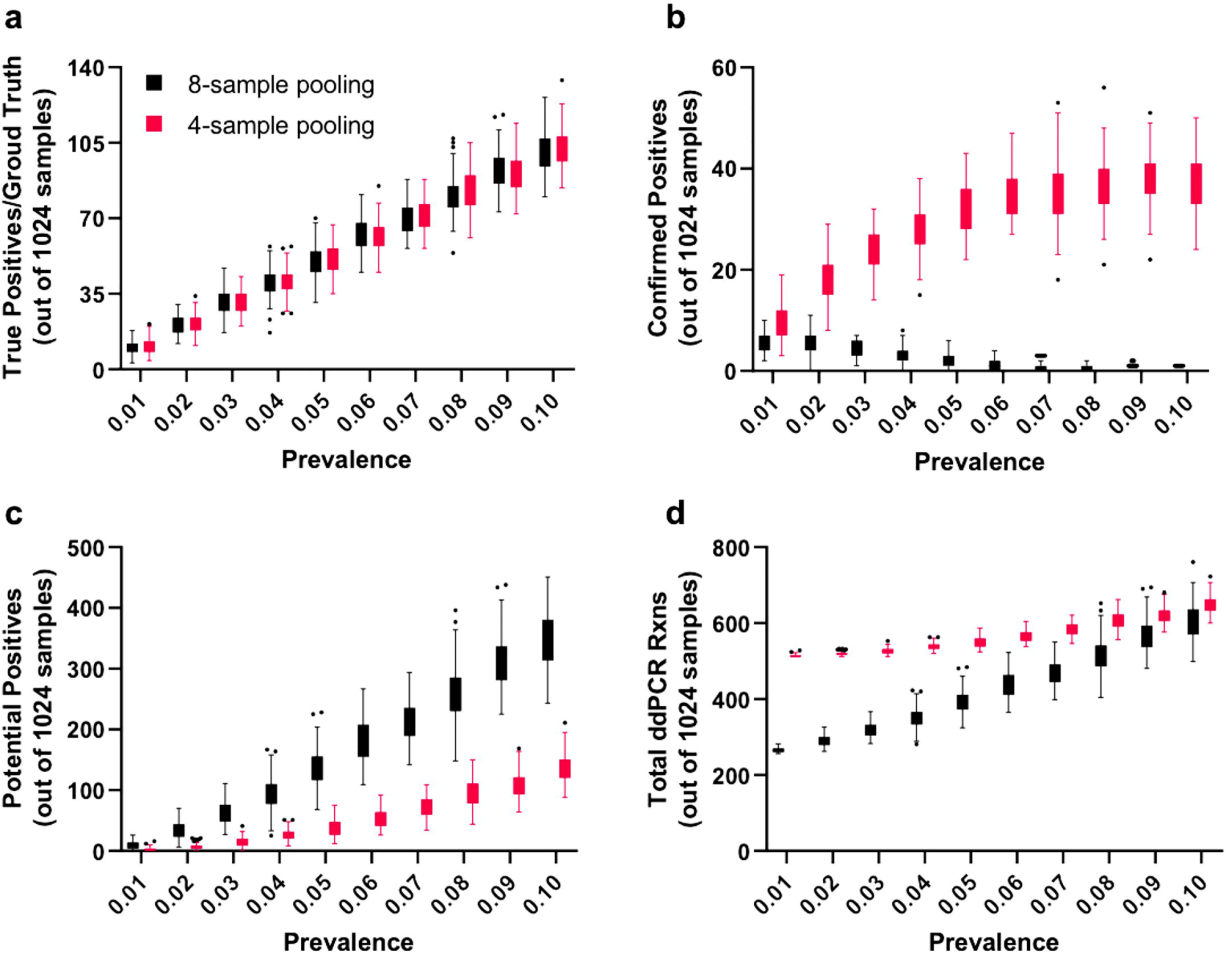
Simulations of 8-sample versus 4-sample pooling strategies. Each simulation contained 1,024 samples arranged in 16, 64-sample plates containing random positive samples at 10 different prevalence values (0.01–0.1). 100 iterations were simulated for each prevalence value and the data summarized using a Tukey boxplot (Graphpad). (**a)** The number of positive samples (ground truth) at each prevalence value. (**b)** The number of positive samples correctly identified by the results from pooled samples. (**c)** The number of potential positive samples identified from the results of pooled samples that would need to be rerun individually for confirmation. (**d)** The total number of ddPCR reactions required to confirm all positives.

We considered several factors when deciding on a pooling strategy, notably the expected positivity rate; cost and availability of reagents, consumables, and personnel; time, and sensitivity. Prior to implementing the MP4 assay, we modeled the number of unambiguously and ambiguously identified positive samples in 8- and 4-sample pooling strategies under positivity rates ranging from 1–10% (Fig. 7). For 8- sample pools, as positivity rate increases the number of positive samples that can be unambiguously identified (confirmed positives) decreases, nearing zero at rates ≥ 6% (Fig. 7b). Concomitantly, the number of potential positives requiring additional testing increased (Fig. 7c). Four-sample pools, on the other hand, required fewer additional tests and were much more resistant to increased positivity rates. Unambiguous positives can be identified at all positivity rates modeled (**Fig. ****7****b**). Prior to implementing our screening assay, we estimated our positivity rates would be < 2% because screening would be limited to an asymptomatic population and because of social distancing and mask requirements instituted on campus. We also chose to implement confirmatory testing of positive samples unambiguously identified from our pooling strategy as a further quality assurance step; the numbers given below include these samples. At a positivity rate of 1%, 4-sample pools would require 515 ± 3 (n = 100) tests (512 pooled tests plus 3 reruns), while 8-sample pools would require 265 ± 7 (n = 100) tests (256 tests plus 9 reruns). At a positivity rate of 2%, 4-sample pools would require 519 ± 5 (n = 100) tests (512 tests plus 7 reruns), while 8-sample pools would require 290 ± 14 (n = 100) tests (256 tests plus 34 reruns) (Fig. 7d). Based on this analysis, we decided to use 8- sample pooling because of the 1.8- 1.9-fold reduction in the number of samples run (resulting in equivalent cost savings) for our expected positivity rate (1–2%). Although 8- sample pooling increased the number of samples requiring reruns, it was not large enough to measurably impact our turnaround time.

### Three-dimensional (3D) pooling

**Figure 8:**
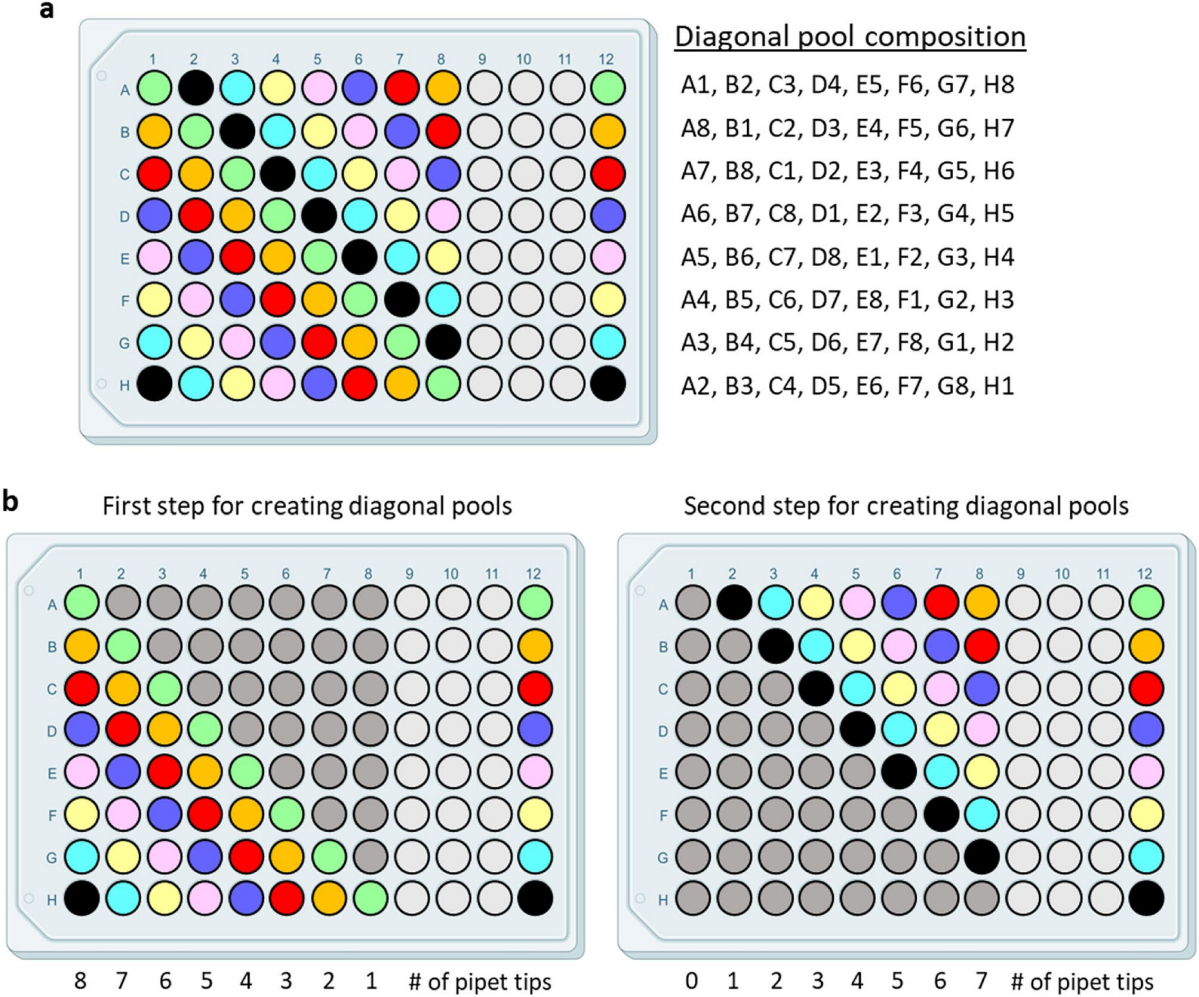
Outline and pipetting strategy for creating diagonal pools**. (a)** We pooled samples diagonally from the indicated wells such that we transferred aliquots of all same-colored samples to their color-matched well in column 12. (**b)** Pipetting strategy to create diagonal pools. In the first step (left), an 8-well multichannel pipettor was loaded with the indicated number of tips. With the pipettor oriented vertically, samples were aliquoted from the individual wells (colored) of each column. Samples were then pipetted into column 12 such that the top-most sample (green) is dispensed into well A12. In the second step, a similar strategy is used, except that samples were pipetted into column 12 such that the bottom-most sample (black) was pipetted into well H12.

**Figure 9:**
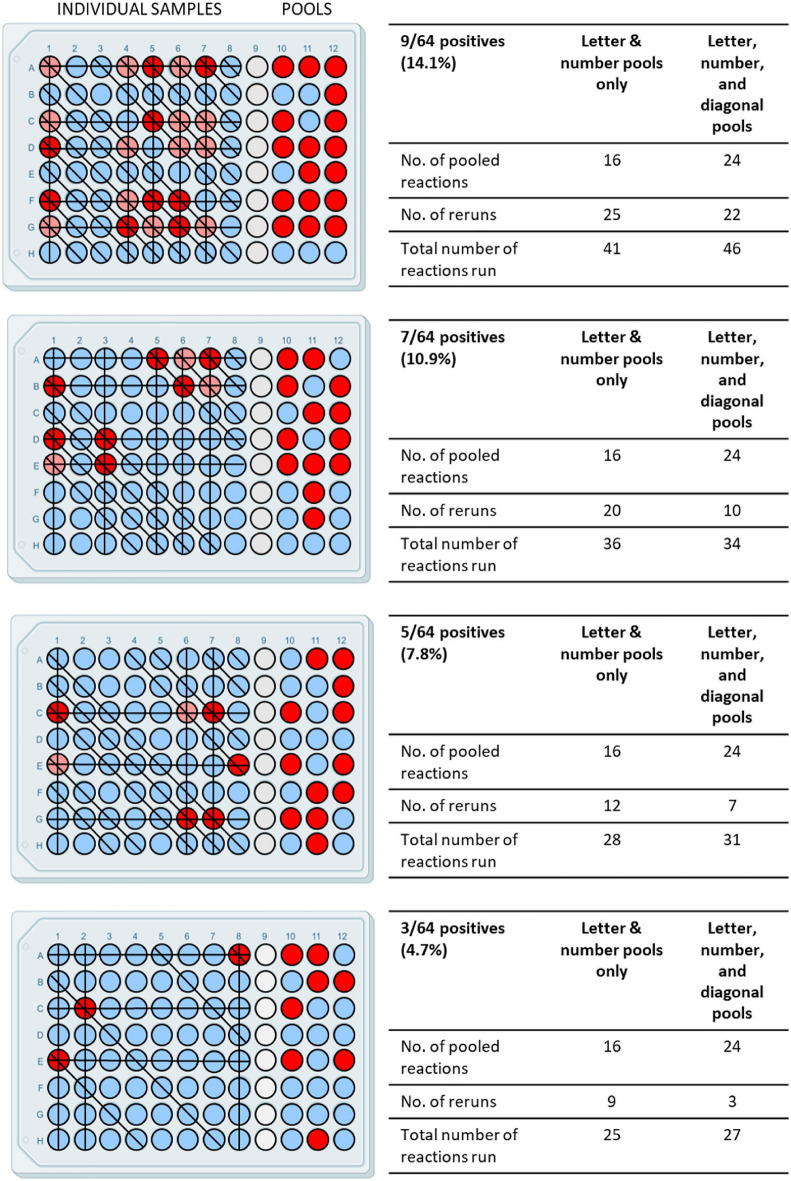
Effect of diagonal pooling on the number of pooled reactions, reruns, and total number of reactions on representative sample plates containing 3, 5, 7, or 9 positive samples out of 64. Individual positive and negative samples are indicated in red and light green, respectively. Potential positives are shown in pink. Positive and negative pooled samples are indicated in red and green, respectively. Columns 10–12 represent letter, number, and diagonal pools, respectively. Inclusion of a third pooled sample increased the number of initial pooled samples from 16 to 24 but reduced the number of rerun reactions.

In addition to reducing pool size, another option for employing the MP4 assay during high disease prevalence is creation of a third pool from the 8 × 8 matrix via diagonal pooling (Fig. 8). Although technically more challenging than creating letter and number pools because of the mechanics of pooling samples oriented on a diagonal (Fig. 8 outlines our pipetting strategy), creation of a third pool can reduce the number of ambiguous samples requiring individual testing (Fig. 9, Supplementary Table s2) and thus turnaround time. Figure 9 presents 4 different sample plate scenarios we observed during the Omicron peak of late December 2021 through January 2022, in which we observed a record number of positive samples per plate (9/64; 14.1% positivity, Supplementary Table s2), and the effect of including a diagonal pool on the number of pooled reactions and reruns. Inclusion of the diagonal pool was very effective at reducing the number of ambiguous samples in plates with ≤ 10% positives. There was minimal effect on the total number of reactions, but the reduction in the number of samples needing reruns would translate to a faster turnaround time.

**Figure 10:**
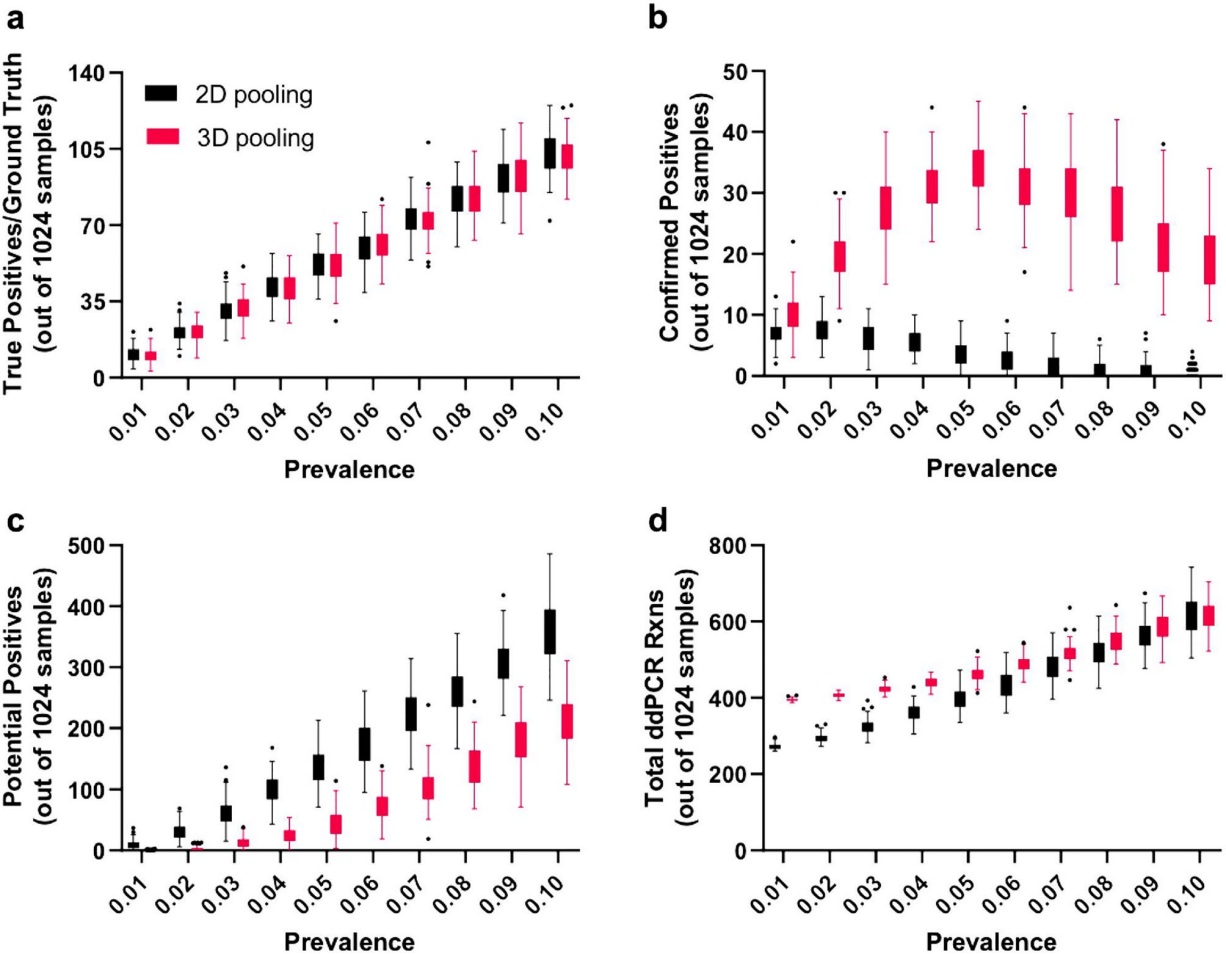
Simulations of 2D and 3D 8-sample pooling strategies**. (a)** Similar sample sets to those in Fig. 7 were created with 1,024 total samples and positive samples randomly introduced at 1–10% positivity rates. (**b, c)** The number of confirmed and potential positives, respectively. Above 8% prevalence no confirmed positive samples can be determined by 2D pooling, while 3D pooling identified significant numbers of known positives even at 10% prevalence. (**d)** As prevalence surpasses 5%, the total number of tests for each strategy equilibrates.

We ran modeling simulations of both 2D and 3D pooling under positivity rates ranging from 1–10% (Fig. 10). Above 8% prevalence, no unambiguous positive samples can be identified in 2D pools (Fig. 10b). The number of ambiguous or “potential” positives is lower for 3D pooling under all positivity rates (Fig. 10c). When prevalence is low (< 6%), the total number of tests required for 3D pooling is higher than that for 2D pooling due to the additional pooled samples (Fig. 10d). However, the total number of tests with 2D and 3D pooling is comparable when positivity rates are 7–9%, and slightly lower for 3D pooling at 10%.

## Discussion

From October 2020 through mid-February 2022, over 240,000 saliva samples were screened using the MP4 assay (Supplementary Fig. s7). There are 3 key features of the MP4 assay, which collectively, distinguish it from other published assays. First, the assay bypasses an RNA extraction step, instead relying on pretreatment of saliva with Proteinase K (PK). This strategy has been used successfully by other groups, although we optimized the MP4 assay to use less PK relative to other published assays to reduce reagent usage and cost (e.g. ~ threefold less than SalivaDirect)^27,28^. Second, we created two paired pools of 8 samples each such that each sample is represented in 2 unique pools. Third, we used ddPCR rather than qPCR as the platform for amplification and detection. Many studies have shown that RT-ddPCR is more sensitive than qPCR for detection of SARS-CoV-2^29–33^, which we reasoned would help offset the reduction in sensitivity with our pooling strategy. ddPCR also is less sensitive to inhibitors due to fractionation of the reaction mixture prior to the RT and amplification steps^34^, which we also reasoned would be advantageous for an assay using minimally processed saliva samples. Relative to the commercially available Bio-Rad SARS-CoV-2 ddPCR kit, we reduced the concentrations of primers and probes to approximately half of what was recommended to conserve on reagents and reduce costs. Since RT-ddPCR was used for COVID screening far less than RT-qPCR, we experienced no supply chain issues or competition for reagents related to the more conventional RT-qPCR tests. Although ddPCR reactions take longer to run than qPCR assays, modification of BioRad’s recommended thermocycler cycling program allowed us to shorten this step from 3.5 to 1.5 h. (Fig. 1, Supplementary Fig. s3). Taken together, these modifications and pooling enabled us to report results the next day for > 95% of samples.

The dataset presented in Fig. 3 highlighted the power of our paired pool approach, by presenting an experiment in which one pool failed to generate a positive call and outlining the resulting steps leading to the successful identification of the positive sample. Pools containing one or more positive samples can result in negative calls for a variety of reasons including low viral loads, the presence of inhibitors, or technical errors such as pipetting. In our data from 2021, 5.5% (n = 130) of our positive samples were identified from a single positive pool, suggesting that while this was a low occurrence, running paired pools increased the detection rate of our assay relative to assays relying on single pools. Most of these samples (88.5%) had < 10 N1 droplets in the corresponding pool and therefore were most likely missed due to low viral concentration. The remaining 11.5% had > 10 N1 droplets in the corresponding pool; technical errors such as pipetting or the presence of inhibitors most likely account for these results.

Overall, our data from August to December of 2021, which included 386 positive samples, showed how well our pooling strategy performed (Fig. 6). The high concordance between the letter and number pools indicated a high degree of technical reproducibility that is remarkable considering this step was performed by > 10 lab personnel within this timeframe. Furthermore, the high degree of correlation between pools and individual samples (~ sevenfold difference) is very close to the predicted eightfold difference based on our pooling strategy as well as the sixfold difference in LOD concentrations observed between individual and pooled samples (2 and 12 copies per µl, respectively). We attribute part of the success of our pooling strategy, as well as the MP4 screening assay, to our reliance on manual processing of saliva samples, avoiding the use of automated liquid handling instruments in favor of manual inspection and pipetting, which allowed greater oversight over all steps of the assay. We and others experienced problems with automated pipetting of saliva due to viscosity and other issues^21^.

Because of the high degree of concordance between the letter and number pools and the quantitative nature of RT-ddPCR, we could often infer which samples were positive in plates with more than one positive sample by “matching” concentrations in the pools. This can be seen in Fig. 5, in which the droplet numbers from the SP5 pools indicated that the positive samples were most likely A5 and D2 because of the similarity in pool droplet numbers (10 and 4 for pools A and 5, respectively and 37 and 35 for pools D and 2, respectively), which was ultimately confirmed in the re-run data. This reveals a further advantage of ddPCR and the MP4 assay that could be exploited to provide enriched, more informative data. Since ddPCR is a quantitative assay that does not require a standard curve, viral loads determined in each reaction can be used to identify potential super shedders or spreaders for prioritization of mitigation efforts to prevent significant community spread. Curiously, we did observe a slightly higher virus titer in number pools. One explanation for these results is that per our standard operating procedure, number pools were created after the letter pools and the reduced volume in the well may have resulted in withdrawal of more of the precipitate in the bottom of the tube. In one study, centrifugation of samples led to a tenfold reduction in LOD, suggesting that the precipitate may harbor a greater concentration of viral material^35^.

Unlike most universities or other entities employing large-scale COVID testing and/or screening, we tested every saliva sample at least in duplicate. Positive samples were confirmed in a third replicate reaction. Thus, the MP4 assay not only saves reagents and costs less than $10 per reaction, but also provides greater assurance that the results are reproducible and reliable.

Although pooling samples has many advantages, especially for screening assays such as ours, one of the biggest limitations is that invalid samples, defined as samples that do not amplify any N1 or RP droplets when run individually, would be called negative in our assay. The threshold for calling a sample negative according to BioRad guidelines is < 2 N1 droplets and ≥ 4 RP droplets, with the presence of RP droplets serving as a necessary internal control to distinguish SARS-CoV-2 negative samples from invalid samples (ex: water). While we monitored RP amplification in our pooled samples, we found it difficult to establish a threshold because of wide variation in RP levels from sample to sample. Furthermore, one or more invalid sample(s) in a pool of 8 would be masked by RP signal from the other samples present in the pool; thus, resulting in those samples being called negative rather than invalid. Because we manually pipetted each saliva sample, we were able to identify potentially problematic samples and flag them for individual testing. However, samples with high levels of inhibitors (tobacco or mouthwash, for instance) may not be distinguishable by eye from valid samples^36^ and could potentially limit the detection of positive samples when present in pools. Our data, however, suggests that pooling acts to dilute inhibitors in samples, as we observed many paired pools that skewed above the trendline shown in Fig. 6 (indicated by green circles), indicating higher than average amplification in pools as compared to the individual sample. Although we believe that this is most likely due to the presence of inhibitors in the individual sample that were diluted during pooling, we also note that there are other explanations for lower-than-expected concentrations in the individual samples such as pipetting errors. Other disadvantages of our assay, such as cost, time, and adherence to a specific platform, all pertain to the RT-ddPCR technology and have been previously reviewed in detail^32^.

Over the course of 311 days of screening (October 2020 to mid-February 2022), we experienced 63 days with positivity rates > 2% (Supplementary Fig. s7). The bulk of these days occurred at the end of 2020 (October-December) and during the Omicron wave at the end of December of 2021 through January of 2022. Although we did not change our pooling strategy during these peak prevalence periods, our modeling data suggests that either shifting to 4-sample pools or including a third pool would have been reasonable strategies (Figs. 7 & 10). 3D pooling would result in fewer pooled samples to test (32 pooled samples per plate for 2D pools/4 samples per pool versus 24 pooled samples for 3D pools/8-samples per pool). In general, any pooling will increase the efficiency of an assay (a measure of the reduction of the number of tests run as compared to running samples individually) (Supplementary Table s2) but will lower the sensitivity (i.e. increase the number of false negatives). As shown here and by others, both smaller pool sizes and 3D pooling are less susceptible to changes in positivity rates^24^. The optimal pooling strategy should be one that is flexible and can be adjusted according to changes in prevalence and viral loads within a population to maximize efficiency and sensitivity.

It is difficult to compare the LOD of assays conducted in different labs using different reference materials, especially given the problems noted by others regarding available reference materials for SARS-CoV-2 assay validation^37–39^. The LOD of the MP4 assay established here (2 copies per µl) is higher than that reported for the BioRad SARS-CoV-2 ddPCR kit (0.15 copies per µL)^40^. This is partly explained by the ~ fourfold increase in volume of starting sample in the BioRad protocol. Our LOD is lower than that reported for SalivaDirect (6–12 SARS-CoV-2 copies per µL)^28^, which has a similar sample workflow, but relies on the less sensitive RT-qPCR platform^41^. Most other screening assays report LODs within this range as well (0.5- 10.6 copies per µl)^16–22^. For pooled samples our LOD was 12 copies per µl, which is in line with at least one other report^20^ and considered an acceptable trade-off for screening assays in which frequency and turnaround time rather than sensitivity are more important for mitigation of viral spread^42^. We propose that individuals with low-titer positive samples likely shed significantly less virus than those with much higher titer samples, whom the MP4 assay easily identifies for isolation before significant spreading of COVID-19 occurs.

The concordance data showed that our assay had very high agreement with an FDA approved ANS test, as we were able to identify all ANS positive individuals as positive with our MP4 assay. We even identified 2 additional individuals as positive that had tested negative via ANS. There are several explanations that could account for the discrepancy between these two tests. First, it is well established that viral loads can differ between sample types (saliva vs ANS) during infection, with viral loads in saliva often detected before those in nares during the early stage of infection^43–46^. Second, inadequate self-collection by ANS could result in a false negative, an issue that is far less encountered with saliva sampling. Third, variant-specific differences related to tissue tropism, as exemplified by the more recent Omicron variants which colonize upper airways and saliva more than lower respiratory and nasal cavities^47,48^.

In our data from August to December of 2021, we noted that the majority of our positive saliva samples had viral loads at the low end of the observed range. This skewed distribution of observed viral loads may be due to our screening of asymptomatic individuals likely in the initial stages of infection during which viral loads and associated shedding are relatively low^49^. This trend has also been observed in other large-scale screening assays^12,19^. These data show that it is possible to detect positive individuals with low viral loads from an asymptomatic population.

Although the MP4 assay was developed in response to the SARS-CoV-2 pandemic, it can be easily adapted for the future. Screening can be easily expanded to other targets of interest and higher order multiplexing for more than 2 targets per reaction can be performed using amplitude and probe-based strategies or with newer ddPCR instrumentation capable of reading more than 2 fluorescent channels^50^. This strategy could prove useful to monitor both COVID-19 and Influenza outbreaks. Although saliva has many advantages as a sample type^51^, we have successfully used the MP4 assay with nasal swabs and non-invasive polyvinyl alcohol (PVA) mask strips (see Reference^52^ for a description of the PVA strips). Depending on the needs of the assay, a wide variety of pooling strategies can be employed to balance sensitivity with efficiency.

## Methods

A graphical overview of the MP4 screening assay is presented in Fig. 1.

### Human subject data

Human subject data was collected in accordance with recruitment and informed consent protocol #20-10256H approved by the Institutional Review Board (IRB) at Colorado State University. All methods were carried out in accordance with relevant guidelines and regulations of the IRB.

### Saliva collection

Five saliva collection sites were set up on the Colorado State University (CSU) campus, including 3 on the main campus and 2 on the more remote South and Foothills campuses (Supplementary Fig. s1). Collection sites were open weekdays for 4–9 h a day, depending on the location. Asymptomatic adults were directed to these saliva collection sites, where they were given a sterile, barcoded 5- or 15- mL conical tube and instructed on saliva collection. Participants were asked to provide 2 ml of saliva. Samples were then transported to the MP4 screening lab where they were immediately placed in a 65 °C water bath for 35 min, previously shown to be effective for inactivation of SARS-CoV-2^53,54^. After inactivation, sample barcodes were scanned and 64 samples were organized into an 8 × 8 matrix whose placement was maintained when transferring to a 96-well microtiter sample plate (SP).

### Sample preparation and pooling

Heat-inactivated saliva was individually pipetted (75 µl) into 96-well microtiter plates containing 9 µl of Proteinase K, Molecular Biology Grade (PK; New England Biolabs) diluted to 267 U/ml in molecular biology grade water. Using PK as an extraction-free method of RNA detection was based on the SalivaDirect protocol^28^ and optimization experiments indicated that 2.4 units of PK was sufficient for reliable RNA detection (data not shown). Plates were then sealed with an adhesive PCR sealing foil sheet (Thermo Scientific, cat no. AB-0626), briefly vortexed, and centrifuged (1 min at 1,210 g). Plates were incubated at 60 °C for 15 min followed by PK inactivation at 95 °C for 5 min. Plates were then centrifuged for 1 min at 1,210 g prior to pooling. A maximum of 64 samples were pipetted per SP in columns 1–8. Columns 10 and 12 were reserved for pooling.

Pools were created with a maximum of 8 samples, using an 8-channel pipettor. First, letter pools were constructed by moving 10 µl of all 8 samples in column 1 into column 10 and subsequently repeating this process for columns 2–8, with new pipette tips employed for each column. Care was also taken to avoid pipetting any particulates pelleted in the prior centrifugation step. The result of this was that well A10 contained samples from row A1-A8; well B10 contained samples from row B1-B8 and so forth (Fig. 1). To construct the number pools, the multichannel pipettor was turned 90° relative to the first pooling step, so that all 8 samples in the A row were pipetted simultaneously. Then, the pipettor was turned another 90° so that the samples were expelled into column 12, with sample A1 being expelled into well A12. The result of this pooling step was that well A12 contained samples from column 1A-H; well B12 contained samples from column 2A-H and so forth. After pooling, plates were sealed with a fresh adhesive sealing foil sheet, mixed by vortexing, and spun for 3 min at 1,210 g prior to RT-ddPCR set-up.

### Reverse transcription droplet digital PCR (RT-ddPCR)

One-Step RT-ddPCR Advanced Kit for Probes (BioRad, cat no. 186–4021) was used for RT-ddPCR according to the manufacturer’s recommendations. Multiplex, 22 µl reactions were set up using 9 µl of pooled saliva sample and nCOV_N1 and RNase P primers and probes developed by the CDC (Division of Viral Diseases, National Center for Immunization and Respiratory Diseases, Centers for Disease Control and Prevention, Atlanta, GA, USA, Supplementary Table s1), although the FAM label on the RNase P probe was replaced with HEX to accommodate multiplexing. Primers and probes were purchased from Integrated DNA Technologies and used at final concentrations of 503 nM and 127.5 nM, respectively. RT-ddPCR reactions were set up in 96-well ddPCR plates (BioRad, cat. No. 12001925) referred to as PCR plates (PP). Plates were covered with a pierceable foil heat seal (BioRad, cat. No. 1814040), sealed using a PX1 PCR Plate Sealer (BioRad, cat. No. 1814000), mixed by vortexing, and centrifuged for 3 min at 1,210 g before droplet generation. Droplets were generated using an Automated Droplet Generator (BioRad), which dispensed droplets into a new 96-well plate. After manual inspection to check for any low or failed droplets, plates were heat-sealed with a pierceable foil and thermocycled on a C1000 touch thermal cycler (BioRad) as follows: 50 °C for 10 min; 95 °C for 10 min; 40 cycles of 94 °C for 10 s and 55 °C for 30 s, followed by a 5 min hold at 4 °C for droplet stabilization. Droplets were then read on a QX200 Droplet Reader (BioRad) set up to read FAM and HEX channels. For additional details on the Bio-Rad RT-ddPCR workflow refer to the protocol by Nyaruaba et al^54^.

### Controls

Four control samples, in duplicate, were included on every RT-ddPCR plate. Controls were prepared daily. The controls were: water samples to monitor for contamination, SARS-CoV-2 negative saliva to monitor RP amplification, and high and low concentrations of a heat-inactivated SARS-CoV-2 virus to monitor N1 amplification. Negative saliva was prepared by pooling heat-inactivated saliva samples from multiple individuals, which was then aliquoted and stored at − 20 °C. Negative saliva was confirmed to be free of SARS-CoV-2 by the Princeton Diagnostic Lab. Negative saliva was prepared by adding 9 µl of 800 U/ml PK (NEB) to 225 µl of negative saliva and incubating as described above. A heat-inactivated SARS-CoV-2 virus, isolate USA-WA1/2020 (NR-52286), was obtained through BEI Resources, NIAID, NIH. Upon receipt, this virus was diluted to 37,500 copies per µl, aliquoted in single use volumes, and stored at -80 °C. A single 10 µl aliquot was thawed on ice, mixed with 5 µl of QuickExtract™ DNA Extraction Solution (QE, Lucigen), and incubated at 65 °C for 6–15 min, followed by 98 °C for 2 minutes^55^. The virus was then diluted with water to 375 copies per µl or 12.5 copies per µl for the high and low positive controls, respectively.

### Data analysis, deconvolution, and reruns

Data analysis was performed in QuantaSoft (BioRad). Using the control samples, thresholds were set approximately twofold above the baseline of the negative droplets. In some cases, thresholds were manually adjusted for individual samples to account for baseline fluctuations and/or increased fluorescence of negative droplets. Samples with < 5,000 accepted droplets were automatically rerun; later this threshold was increased to 10,000 droplets. Samples were considered positive for SARS-CoV-2 if they had at least 3 positive droplets for N1^54^. After deconvolution of the positive pool(s), individual samples from the SPs were rerun to confirm the positive samples. Samples were identified as either positive or negative for SARS-CoV-2. Additionally, samples were called invalid if saliva sample was not present, could not be pipetted, was flagged for qualitative reasons, or did not meet our QC threshold for RP. Our invalid rate was 0.13%.

### Limit of detection (LOD)

LOD studies were determined using 3 different SARS-CoV-2 virus sources. A heat-inactivated SARS-CoV-2 virus isolate, described above, was extracted by adding QE solution to virus in a 1:2 ratio and incubating at 65 °C for 15 min, followed by 98 °C for 2 min. The viral concentration in this tube after QE extraction was predicted to be 2.5 × 10^4^ copies per µl based on copy numbers provided by the manufacturer. In initial range finding experiments, viral extracts were serially diluted tenfold in sterile water in triplicate. Further dilutions at 1, 2, 3, and 4 copies per µl were performed with 20 replicates each to establish the LOD. Viral RNA was extracted from the heat-inactivated SARS-CoV-2 virus isolate using the QIAmp Viral RNA Mini kit (BioRad) according to the manufacturer’s guidelines. Viral RNA was serially diluted twofold in water and quantified using RT-ddPCR^38^. Linear regression of the data (spanning 10 dilutions) demonstrated excellent linearity (R^2^ = 0.9994) and based on this data, we assigned the sample a concentration of 72,486 copies per µl. LOD was determined as described above, by diluting the viral RNA in water. Quantification of a pooled positive saliva sample was performed by first extracting the viral RNA using PK, followed by serial dilutions in water and quantification by RT-ddPCR. Sample concentration was determined to be 5,645 copies per µl. LOD was determined by spiking this positive saliva sample into pooled negative saliva, performing serial dilutions in negative saliva, followed by PK extraction and RT-ddPCR. For determination of the LOD for pooled samples, the positive saliva sample was diluted in negative saliva and added to the first column of a 96 well plate. Negative saliva was added to columns 2–8. After PK digestion, samples (10 µl each) were pooled vertically using an 8-channel pipetter into column 10. RT-ddPCR on the pooled samples was as described above.

### Pooling simulations and deconvolution

A simulation was performed in Matlab (Ver. 2019b)^56^ by creating 16 virtual sample plates, each with a total of 64 samples arranged into an 8 × 8 grid as described for the MP4 assay. Each sample was randomly assigned as positive (1) or negative (0) based on the positivity rate (0.01–0.1) for that iteration of the simulation. Samples were virtually pooled according to the MP4 method in either 8-sample or 4-sample pools by summing the rows and columns such that each pool had a numerical value. The numerical values of each pool were converted to a binary readout, either positive (≥ 1) or negative (0). Deconvolution was performed using only the binary readout for each pool, similar to how the MP4 assay is conducted. The simulation was used to predict the number of individual samples for retesting, using only the results from the pooled samples to instruct the deconvolution. The simulation was run 1,000 times, with 100 iterations for each prevalence level (0.01–0.1). Results are reported as a boxplot using the Tukey method (Graphpad) for 100 iterations of the simulation. Simulations of 2D and 3D sample pooling and deconvolution were written in R version 4.1.2 and used the same approach as described above, but with the addition of a third set of pools.

## Supplementary Information

Supplementary Information.

## Data Availability

The datasets used and/or analyzed during the current study available from the corresponding author on reasonable request.
