# Author Correction: A multiplexed, paired-pooled droplet digital PCR assay for detection of SARS-CoV-2 in saliva

**DOI:** 10.1038/s41598-024-52768-z

**Published:** 2024-02-07

**Authors:** Kaitlyn Wagner, Phil Fox, Elizabeth Gordon, Westen Hahn, Kenzie Olsen, Alex Markham, Dylan Buglewicz, Platon Selemenakis, Avery Lessard, Daniella Goldstein, Alissa Threatt, Luke Davis, Jake Miller-Dawson, Halie Stockett, Hailey Sanders, Kristin Rugh, Houston Turner, Michelle Remias, Maggie Williams, Jorge Chavez, Gabriel Galindo, Charlotte Cialek, Amanda Koch, Alex Fout, Bailey Fosdick, Bettina Broeckling, Mark D. Zabel

**Affiliations:** 1 Prion Research Center, Department of Microbiology, Immunology and Pathology, College of Veterinary Medicine and Biomedical Sciences, Fort Collins, USA; 2 Department of Statistics, Colorado State University, Fort Collins, CO 80523 USA; 3 Colorado State University, Fort Collins, CO 80523 USA; 4 Colorado School of Public Health, University of Colorado Anschutz Medical Campus, Aurora, USA

Correction to: *Scientific Reports* 10.1038/s41598-023-29858-5, published online 22 February 2023

Hailey Sanders was omitted from the author list in the original version of this Article.

The Author Contributions section now reads:

 “K.W., P.F., E.G., and M.D.Z. conceived of the MP4 assay. P.F, A.F., and B.F. performed the modeling simulations. K.W., K.O., B.B. and M.D.Z. wrote the manuscript. H.T. and M.R. organized IRB recruitment and consent. K.W., P.F., E.G., W.H., A.M., K.O., D.B., P.S., A.L., D.G., A.T., L.D., J. M.-D., H.S., H.S., K.R., M.W., J.C., G.G., C.C., A.K., B.B. and M.D.Z. developed, optimized and performed the assay, collected data and provided critical feedback on the manuscript.”

The original Article has been corrected.

