# Supplementary material for: A multiplexed, paired-pooled droplet digital PCR assay for detection of SARS-CoV-2 in saliva
Source: Sci Rep. 2023 Feb 22;13:3075. doi: 10.1038/s41598-023-29858-5 (PMC9944410; doi:10.1038/s41598-023-29858-5)
Supplement: Supplementary file 1 — Supplementary Information. [file 41598_2023_29858_MOESM1_ESM.docx]

**Supplementary Information for**

**A multiplexed, paired-pooled droplet digital PCR assay for detection of SARS-CoV-2 in saliva**

Wagner *et al*

**Supplementary Figures**

**
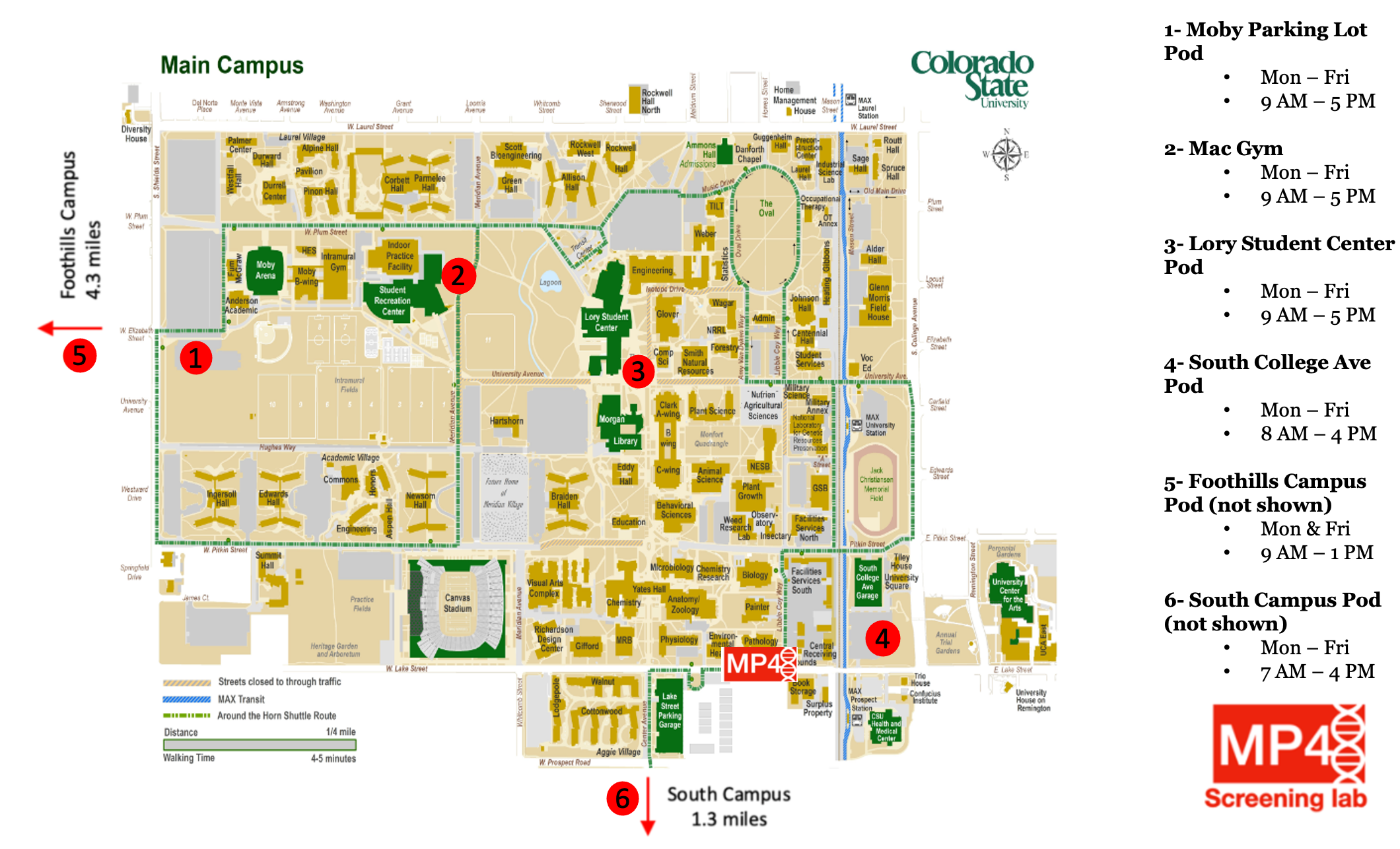
Supplementary Fig. 1. Map of Colorado State University main campus showing locations of saliva collection sites and MP4 saliva screening lab*.*** Locations of the three saliva screening sites on the main campus are numbered 1-4. The Mac gym facility (2) was the original collection site from October 1, 2020 until May 27, 2021. The collection pods opened in May, 2021. The saliva screening lab was in the Pathology building. Foothills (5) and South Campus (6) pods were located on satellite campuses 4.3 and 1.3 miles from main campus, respectively. Hours of operation of the five saliva screening sites are displayed. Sites were closed on weekends, university recognized holidays, and during inclement weather. This map was modified with permission from CSU from <https://catalog.colostate.edu/general-catalog/welcome/map/> .


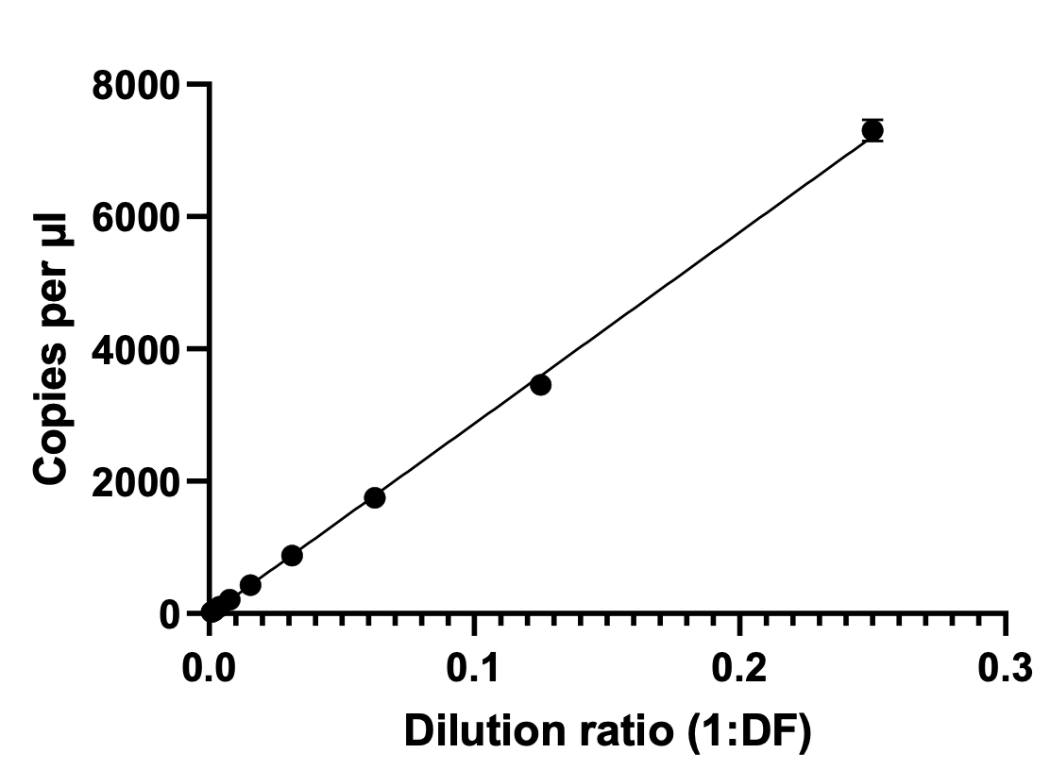


**Supplementary Fig. 2. Linearity of the MP4 assay**. Linearity was determined by plotting the concentration (copies/μl in the RT-ddPCR reaction) of serial dilutions of viral RNA diluted in water. Linear range was determined to be 0.29– 7,300 copies/μl (R^2^: 0.9990).

|  | **MON** | **TUE** | **WED** | **THU** | **FRI** | **SAT** | **SHIFT DUTIES** |  |
| --- | --- | --- | --- | --- | --- | --- | --- | --- |
| **REURNS 6 AM - 12 PM** | N/A | 1 RA | 1 RA | 1 RA | 1 RA | 1-2 RAs | Data analysis and positive sample confirmation; Send test results to PPT |  |
| **MORNING 8 AM- 12PM** | 3 RAs 1 LA | 3 RAs 1 LA | 3 RAs 1 LA | 3 RAs 1 LA | 3 RAs 1 LA | N/A | PK plate and Control prep; Sample intake and processing; trash disposal and cleaning |  |
| **AFTERNOON 12 PM - 5 PM** | 5 - 7 RAs | 5 - 7 RAs | 5 - 7 RAs | 5 - 7 RAs | 5 - 7 RAs | N/A | Sample intake and processing; Pooling; ddPCR Master Mix prep, droplet generation and PCR |  |
| **EVENING 5 PM - 10 PM** | 2 - 3 RAs 1 LA | 2 - 3 RAs 1 LA | 2 - 3 RAs 1 LA | 2 - 3 RAs 1 LA | 2 - 3 RAs 1 LA | N/A | Sample intake and processing; Pooling; ddPCR Master Mix prep, droplet generation and PCR; Droplet reading; Data analysis; Sample and data organization and storage; cleaning and decontamination tasks |  |
| RA, Research Associate; LA, Laboratory Assistant; PPT, Pandemic Preparedness Team | | | | | | | | |

**Supplementary Fig. 3. Typical Schedule and Shift Workflow for the MP4 SARS-CoV-2 Saliva Screening Lab**. Monday through Friday the MP4 lab was typically staffed from morning (as early as 6 am) to evening (11 pm or later), with the number of staff required during the day indicated. Morning shifts were typically spent performing data analysis and confirmatory testing of the previous day’s samples (reruns) as well as preparing for the current day’s samples (preparation of ~40 PK plates and controls). Saliva samples were brought to the lab throughout the day, starting around 9 AM, and processing began immediately starting with heat inactivation. After 5 Sample Plates were processed and pooled (by 2 PM, on average), set up of PCR plates began followed by droplet generation, thermocycling, and droplet reading. During instrument wait times in the evening, staff performed lab upkeep (cleaning, restocking) and data management.


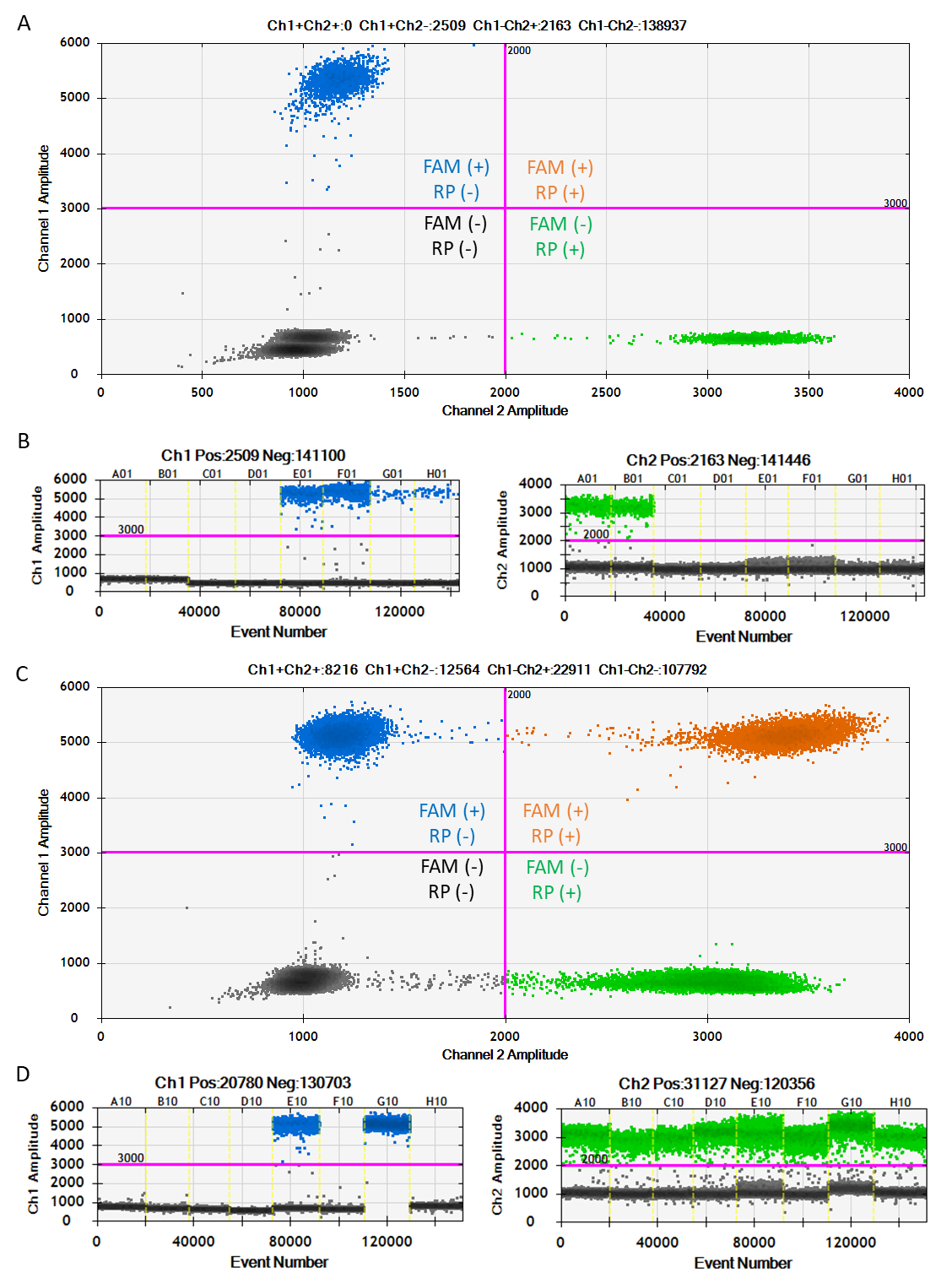
**Supplementary Fig. 4. Representative example of droplet reader results for control (A & B) and pooled (C & D) samples**. **A, C.** Amplitude plots of droplets with HEX (RP) fluorescence plotted on the X-axis and FAM (N1) fluorescence plotted on the y-axis for control **(A)** and pooled **(C)** samples. Thresholds are indicated by pink lines. **B, D**. FAM (blue) and HEX (green) fluorescence amplitudes are plotted for each sample. **B.** Samples are SARS-CoV-2 pooled negative saliva (A01&B01), water (C01&D01), and high (E01&F01) and low (G01&H01) SARS-CoV-2 controls. **D.** Samples represent a column of pooled samples. Note sample in well G10 has no negative N1 droplets.


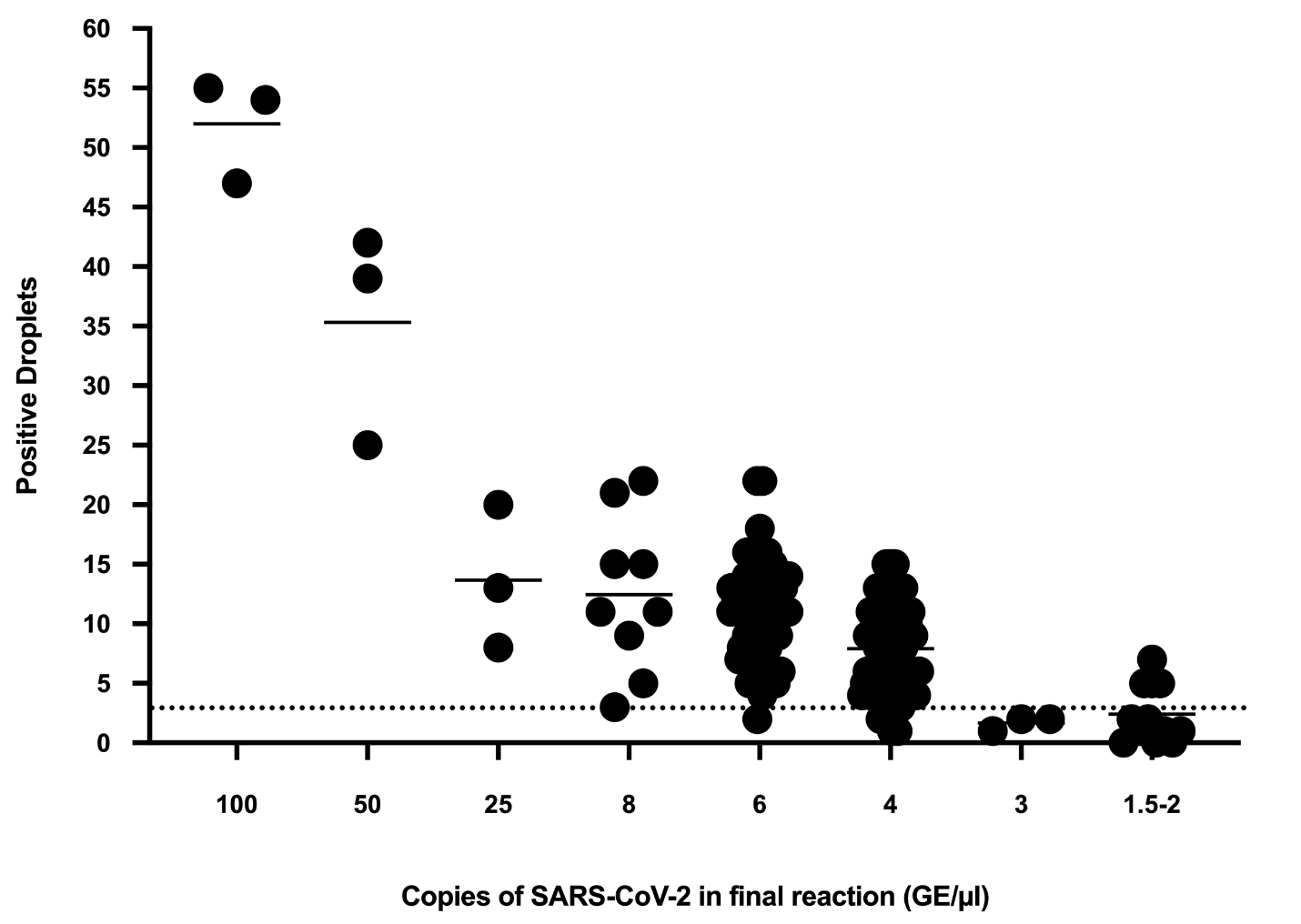
**Supplementary Fig. 5. LOD experiments using heat-inactivated virus from BEI Resources.** LOD was determined by spiking heat-inactivated virus from BEI Resources into SARS-CoV-2 negative saliva, followed by PK digestion, and RT-ddPCR. The LOD was determined to be 11 SARS-CoV-2 copies per µl. Data points above the dotted line (set at 3 copies per µl) meet our threshold of ≥3 positive N1 droplets. LOD is represented as SARS-CoV-2 copies per µl in the starting sample and was defined as the lowest concentration of virus in which ≥95% of replicates (n≥20) were scored as positive.

 **Supplementary Fig. 6**. Histogram showing the frequency of SARS-CoV-2 copies per reaction in tests from positive individual samples from Fall semester 2021 (n=386). Numbers on the x-axis represent the center values for bins with a width of 10,000 copies per reaction. Y-values are plotted on a log_2_ scale for clarity. Mean and median are 9,440 and 591 copies per reaction, respectively.


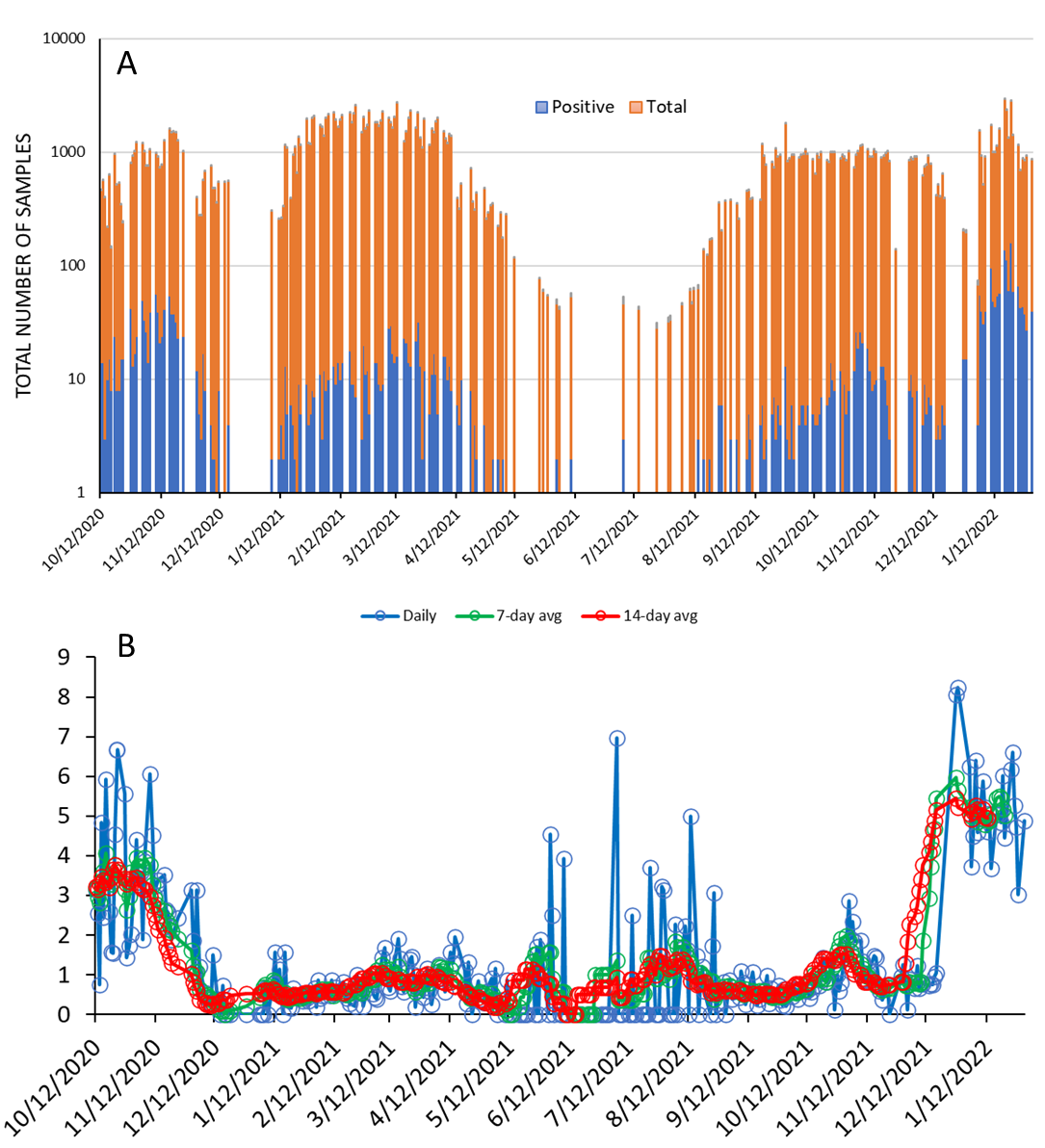
**Supplementary Figure 7. Number of saliva tests and positivity rate per day of saliva screening. A.** The total (orange) and positive (blue) number of saliva samples screened by the MP4 assay. The y-axis is plotted on a log_10_ scale. **B**. Daily (blue) percent positivity and 7-day (green) and 14-day (red) averages for the the MP4 screening assay.

**Supplementary Tables**

**Supplementary Table 1. Primer and Probe Sequences for N1 and RP.**

| **Label** | **Description** | **Sequence** | **Label** |
| --- | --- | --- | --- |
| 2019-nCoV_N1-F | N1 forward primer | GACCCCAAAATCAGCGAAAT | None |
| 2019-nCoV_N1-R | N1 reverse primer | TCTGGTTACTGCCAGTTGAATCTG | None |
| 2019-nCoV_N1-P | N1 probe | FAM-ACCCCGCAT /ZEN/ TACGTTTGGTGGACC-3IABkFQ | FAM^1^, ZEN^2^, 3IABkFQ^3^ |
| RP-F | RNase P forward primer | AGATTTGGACCTGCGAGCG | None |
| RP-R | RNase P reverse primer | GAGCGGCTGTCTCCACAAGT | None |
| RP-P | RNase P probe | HEX/ TTC TGA CCT /ZEN/ GAA GGC TCT GCG CG /3IABkFQ/ | HEX^4^, ZEN^2^, 3IABkFQ^3^ |

^1^FAM: 6-carboxyfluorescein

^2^ZEN: ZEN™ Internal Quencher

^3^3IABkFQ: Iowa Black® FQ

^4^HEX: Hexachloro-fluorescein

**Supplementary Table 2. Impact of pooling strategy on the number of reactions needed to unambiguously identify positive samples under the 4 different scenarios presented in Fig. 9**.

| **9/64 positives (14.1%)** |  | **No pooling** | **Letter pool** | **Number pool** | **Diagonal pool** | **Letter & number pools (2D)** | **Letter, number, & diagonal pools (3D)** |
| --- | --- | --- | --- | --- | --- | --- | --- |
|  | **No. of pooled reactions** |  | 8 | 8 | 8 | 16 | 24 |
|  | **No. of reruns** |  | 40 | 40 | 56 | 25 | 22 |
|  | **Total no. of reactions run** | 64 | 48 | 48 | 64 | 41 | 46 |
| **7/64 positives (10.9%)** |  |  |  |  |  |  |  |
|  | **No. of pooled reactions** |  | 8 | 8 | 8 | 16 | 24 |
|  | **No. of reruns** |  | 32 | 40 | 32 | 20 | 10 |
|  | **Total no. of reactions run** | 64 | 40 | 48 | 40 | 36 | 34 |
| **5/64 positives (7.8%)** |  |  |  |  |  |  |  |
|  | **No. of pooled reactions** |  | 8 | 8 | 8 | 16 | 24 |
|  | **No. of reruns** |  | 24 | 32 | 40 | 12 | 7 |
|  | **Total no. of reactions run** | 64 | 32 | 40 | 48 | 28 | 31 |
| **3/64 positives (4.7%)** |  |  |  |  |  |  |  |
|  | **No. of pooled reactions** |  | 8 | 8 | 8 | 16 | 24 |
|  | **No. of reruns** |  | 24 | 24 | 16 | 9 | 3 |
|  | **Total no. of reactions run** | 64 | 32 | 32 | 24 | 25 | 27 |
